# Specialized Motor-Driven dusp1 Expression in the Song Systems of Multiple Lineages of Vocal Learning Birds

**DOI:** 10.1371/journal.pone.0042173

**Published:** 2012-08-02

**Authors:** Haruhito Horita, Masahiko Kobayashi, Wan-chun Liu, Kotaro Oka, Erich D. Jarvis, Kazuhiro Wada

**Affiliations:** 1 Department of Neurobiology, Howard Hughes Medical Institute, Duke University Medical Center, Durham, North Carolina, United States of America; 2 School of Fundamental Science and Technology, Keio University, Yokohama, Kanagawa, Japan; 3 Department of Biological Sciences, Faculty of Science, Hokkaido University, Sapporo, Hokkaido, Japan; 4 School of Life Science, Hokkaido University, Sapporo, Hokkaido, Japan; 5 Field Research Center, The Rockefeller University, Millbrook, New York, United States of America; Rutgers University, United States of America

## Abstract

Mechanisms for the evolution of convergent behavioral traits are largely unknown. Vocal learning is one such trait that evolved multiple times and is necessary in humans for the acquisition of spoken language. Among birds, vocal learning is evolved in songbirds, parrots, and hummingbirds. Each time similar forebrain song nuclei specialized for vocal learning and production have evolved. This finding led to the hypothesis that the behavioral and neuroanatomical convergences for vocal learning could be associated with molecular convergence. We previously found that the neural activity-induced gene dual specificity phosphatase 1 (dusp1) was up-regulated in non-vocal circuits, specifically in sensory-input neurons of the thalamus and telencephalon; however, dusp1 was not up-regulated in higher order sensory neurons or motor circuits. Here we show that song motor nuclei are an exception to this pattern. The song nuclei of species from all known vocal learning avian lineages showed motor-driven up-regulation of dusp1 expression induced by singing. There was no detectable motor-driven dusp1 expression throughout the rest of the forebrain after non-vocal motor performance. This pattern contrasts with expression of the commonly studied activity-induced gene egr1, which shows motor-driven expression in song nuclei induced by singing, but also motor-driven expression in adjacent brain regions after non-vocal motor behaviors. In the vocal non-learning avian species, we found no detectable vocalizing-driven dusp1 expression in the forebrain. These findings suggest that independent evolutions of neural systems for vocal learning were accompanied by selection for specialized motor-driven expression of the dusp1 gene in those circuits. This specialized expression of dusp1 could potentially lead to differential regulation of dusp1-modulated molecular cascades in vocal learning circuits.

## Introduction

**Figure 1:**
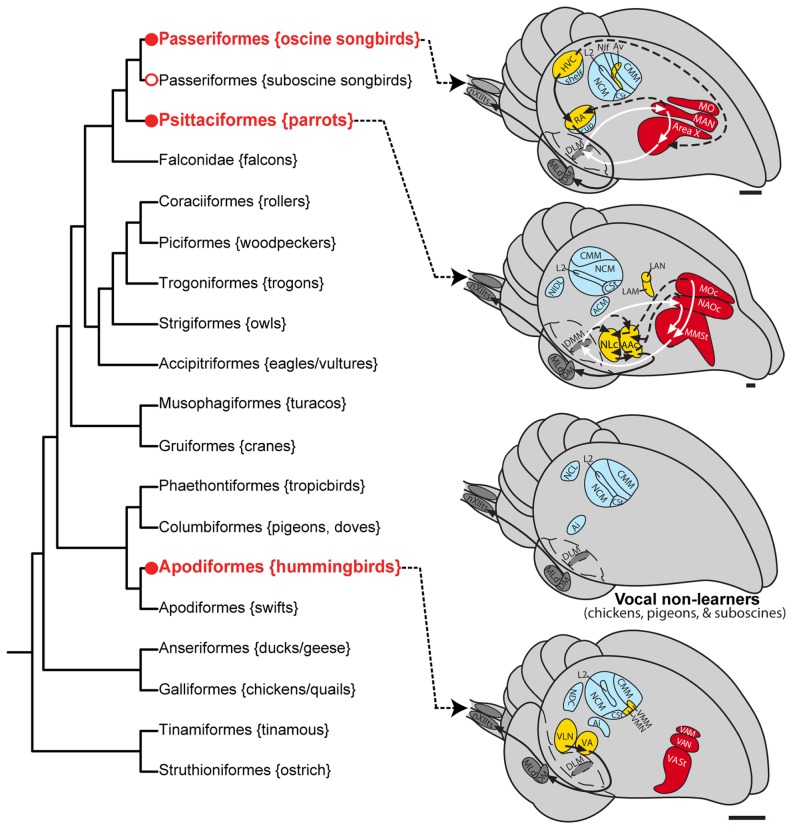
Phylogenetic relationships and vocal pathways in avian vocal learners and vocal non-learners. Left: Phylogeny of some of the major avian orders based on DNA sequences of 19 nuclear loci [14] leads to our suggestion of two independent gains (hummingbirds and ancestor of parrots and oscine songbirds) and then a lost in suboscine songbirds. Also see [15] for support of this view. Alternative phylogenies exist, all with vocal learners distantly related to each other [106], [107]. This phylogenetic tree should be treated as a hypothesis as it is subject to change with more DNA sequences added. The Latin name of each order is given, along with examples of common species. Circles show the minimal ancestral nodes where vocal learning could have either evolved (red) or been lost (white) independently. Right: Proposed comparable vocal and auditory brain areas among vocal learning and vocal non-learning birds. Yellow regions and black arrows, posterior vocal pathways; red regions and white arrows, anterior vocal pathways; dashed lines, connections between the two vocal pathways; blue, auditory regions. For simplification, not all connections are shown. The thalamus has broken-line boundaries to indicate that it is covered by the telencephalon in this view. Not all species have been examined for the presence or absence of song nuclei. Neuroanatomical data of representative species are from the following publications [28], [29], [69], [90], [108]. Scale bars ≈ 1 mm. Abbreviations: ACM, caudal medial arcopallium; NCL, caudal lateral nidopallium; NDC, caudal dorsal nidopallium; NIDL, dorsal lateral intermediate nidopallium. For other anatomical abbreviations, see Table 1.

Characterizing the molecular basis for the evolution of convergent traits may help us to understand the genetics of adaptation. This question has received increasing attention [1]–[3]. Examples of convergent genetic changes contributing to convergent traits include *cis*-regulatory or protein coding mutations in: i) a pigmentation gene that generates similar but independently evolved wing spots in multiple fruit fly species [4]; ii) a homeobox transcription factor that leads to pelvic reduction in independent lineages of both stickleback fish and mammals [5]; iii) a sodium channel gene that was important for the evolution of electric organs in independent lineages of electric fish [6]; and iv) the melanocortin 1 receptor gene responsible for coat and skin color variation across vertebrates [2]. All of these examples are traits involving a convergent phenotype that is easily visible in the organism. In contrast, vocal learning is a complex behavioral trait that is not easily visible but shows striking convergent evolution. Vocal learning includes the ability to imitate sounds and culturally transmit vocal repertoires from one generation to the next [7]–[9]; it is essential for spoken language acquisition [10]. Vocal learning is found in only a few groups of distantly related mammals (humans, cetaceans, bats, in two individual elephants, and possibly pinnepeds) and a few groups of distantly related birds (oscine songbirds, parrots, and hummingbirds) [8], [11], [12]. Because this trait is not found in species closely related to each vocal learning lineage, some researchers have argued that vocal learning evolved independently in each lineage [8], [13]. Recent phylogenetic analyses of 19 genes and several retrotranposons suggest that parrots may be more closely related to songbirds than previously thought [14], [15]. These findings led to the novel proposal that vocal learning evolved twice in birds (once in hummingbirds and again in the common ancestor of songbirds and parrots) and was subsequently lost in suboscine songbirds (**Fig. 1**). However, this interpretation depends on the genes used and whether protein or nucleotide data are examined [16]. In either case, it is generally agreed the both songbirds and parrots are distantly related to hummingbirds.

Despite their possible independent origins of vocal learning, all vocal learning birds have song pathways composed of seven forebrain song nuclei with similar, although not identical, topology, anatomy, function, and connectivity (**Fig. 1**; [11], [17]–[22]; **Table 1** lists all anatomical regions studied and their relative similarities across species). In songbirds, the most well-studied group, these nuclei are distributed into two sub-pathways: i) a posterior vocal pathway that connects the forebrain to brainstem vocal nuclei, is similar to mammalian motor pathways, and is critical for the production of learned vocalizations; and ii) an anterior vocal pathway that forms a pallial-basal ganglia-thalamic loop, is similar to such loops in mammalian brains, and is necessary for song learning (**Fig. 1**; [11], [17], [23]–[26]). None of these song nuclei have been found to date in vocal non-learning species, even in suboscine songbirds, which are closely related to oscine songbirds, or in ring doves (relative of pigeons), which are considered a close sister branch to the group that includes hummingbirds (**Fig. 1**; [27]–[30]; to prevent confusion, we refer to oscine songbirds as 'songbirds' and suboscine songbirds as 'suboscine birds' [28]). Anatomical, molecular neurochemical, developmental, and behavioral evidence show that both vocal learners and vocal non-learners possess homologous brainstem vocal nuclei for production of innate vocalizations (these nuclei also participate in the production of learned vocalizations in vocal learners) and an auditory forebrain pathway used for auditory processing and learning (**Fig. 1**; [11], [31], [32]).

**Table 1:** Terminology of comparable brain areas of avian vocal learners.

Modality	Vocal			Movement			Auditory
Species	Song	Parrot	Humb	Song	Parrot	Humb	All
Subdivision							
**Nidopallium**	HVC	NLc	VLN	DLN	SLN	DLN	L1, L2,
	NIf	LAN	VMN	PLN	LN	n.d.	L3, NCM
	MAN	NAO	VAN	AN	AN	AN	
**Mesopallium**	Av	LAM	VMM	PLMV	LMV	n.d.	CM
	MO	MO	VAM	AMV	AMV	AMV	
**Arcopallium**	RA	AAc	VA	LAI	LAI	LAI	AI
**Striatum**	Area X	MMSt	VASt	ASt	ASt	ASt	CSt
**Thalamus**	aDLM	DMM	aDLM	DLM			Ov
	Uva						
**Midbrain**	DM	DM	DM				MLd

Song, songbird. Humb, hummingbird. n.d., not done. Abbreviations are listed below.

**Abbreviations**

AAc, central nucleus of the anterior arcopallium

aDLM, anterior nucleus of DLM

AI, intermediate arcopallium

AMV, anterior ventral mesopallium

AN, anterior nidopallium

Area X, a vocal nucleus (no acronym)

ASt, anterior striatum

Av, nucleus avalanche

CM, caudal mesopallium

CMM, caudal medial mesopallium

CSt, caudal striatum

DLM, dorsal lateral nucleus of the medial thalamus

DM, dorsal medial nucleus of the midbrain

DMM, magnocellular nucleus of the dorsal thalamus

HVC, a vocal nucleus (no acronym)

LAI, lateral intermediate arcopallium

LAM, lateral nucleus of the anterior mesopallium

LAN, lateral nucleus of the anterior nidopallium

LMV, lateral ventral mesopallium

LN, lateral nidopallium

MAN, magnocellular nucleus of the anterior nidopallium

MLd, dorsal part of the lateral mesencephalic nucleus

MMSt, magnocellular nucleus of the medial striatum

MO, oval nucleus of the mesopallium

NAO, oval nucleus of the anterior nidopallium

NCM, caudal medial nidopallium

NIf, interfacial nucleus of the nidopallium

NLc, central nucleus of the lateral nidopallium

nXIIts, 12^th^ nucleus, tracheosyringeal part

Ov, nucleus ovoidalis

PLMV, posterior lateral ventral mesopallium

PLN, posterior lateral nidopallium

RA, robust nucleus of the arcopallium

SLN, supra lateral nidopallium

Uva, Nucleus Uvaeformis

VA, vocal nucleus of the arcopallium

VAM, vocal nucleus of the anterior mesopallium

VAN, vocal nucleus of the anterior nidopallium

VASt, vocal nucleus of the anterior striatum

VLN, vocal nucleus of the lateral nidopallium

VMM, vocal nucleus of the medial mesopallium

VMN, vocal nucleus of the medial nidopallium

The song nuclei and adjacent areas have similar activity-dependent molecular responses, showing, respectively, robust singing-driven and movement-driven expression of both immediate early genes (IEGs) c-fos and egr1 (a.k.a. zif268, NGF-1A, Krox-24, and ZENK; [29]). These IEGs are transcription factors that regulate expression of other genes involved in synaptic and behavioral plasticity [33]. We recently discovered that another IEG, dusp1 (also known as mitogen-activated protein kinase (MAPK) phosphatase 1 [mkp1]), has a complementary regulatory pattern to egr1 and c-fos [34]. Dusp1 expression is induced by sensory stimuli in the sensory input neurons of the thalamus and telencephalon, where egr1 expression is not induced by neural activity. Conversely, egr1 expression is induced by neural activity in higher order sensory neurons and in motor regions adjacent to song nuclei, where dusp1 expression is not induced [34]. Consistent with these findings, high levels of behaviorally-driven dusp1 and egr1 were not found in the same neurons [34]. These findings supported the hypothesis based on data from *in vitro* studies that dusp1 is a potent suppressor of egr1 expression [35]. Dusp1 is an important regulator of signal transduction pathways in cells and acts by dephosphorylating the MAPK family of proteins, thereby affecting induction of downstream genes [35], [36].

Here we report that, in contrast to other motor systems, dusp1 showed robust motor-driven, singing-induced expression in song nuclei of species from all three vocal learning lineages. For the songbird species examined, high levels of dusp1 and egr1 were almost exclusively co-expressed in the same neurons within song nuclei but not in the rest of the brain. We found no evidence of vocalizing-induced dusp1 expression in the forebrain of vocal non-learners [37]. These findings suggest that selection for specialized motor-driven expression of the dusp1 gene in vocal learning circuits occurred during the evolution of brain systems for vocal learning in multiple lineages of avian vocal learners.

## Results

### Dusp1 is regulated by singing in song nuclei of a songbird

**Figure 2:**
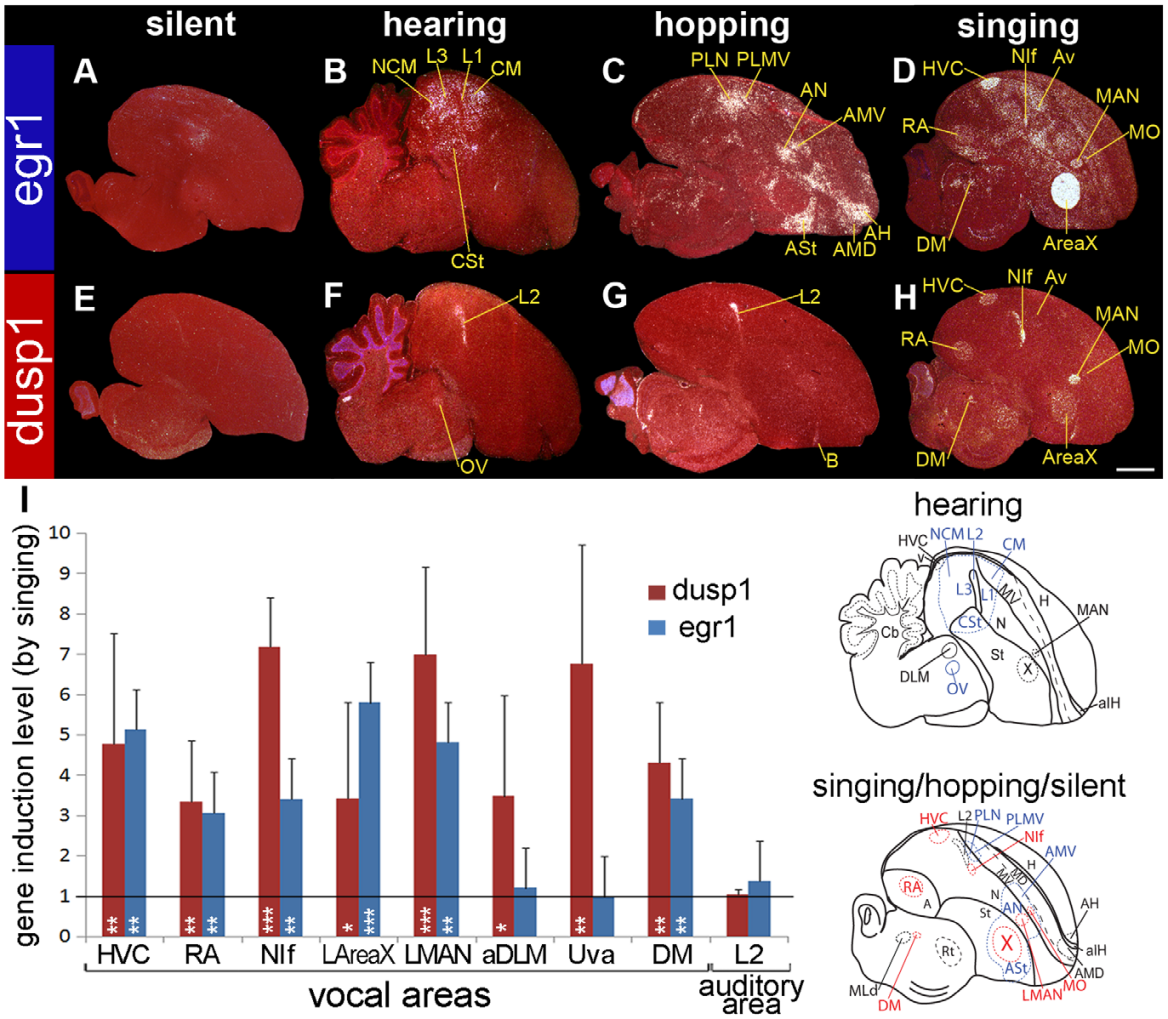
Egr1 and dusp1 mRNA expression in zebra finch brain induced by hearing, hopping, and singing. (**A–D**) Darkfield images of *in situ* hybridizations with egr1 from male zebra finches of four different behavioral conditions: (**A**) silent control sitting in the dark; (**B**) sitting and hearing song for 30 min in the dark; (**C**) deaf animals hopping in a rotating wheel in the dark; and (**D**) singing alone (>305.6 sec; >102 song bouts) and some hopping for 30 min in the light. (**E–H**) Adjacent sagittal sections hybridized to dusp1. All animals were in sound attenuation chambers. Three regions show overlap of hearing-driven and movement-driven gene expression: egr1 in PLN and PLMV, and dusp1 in the adjacent part of L2. See [29], [34] for more details on hearing- and movement-driven gene expression results. Song nuclei are the only areas with overlap in induced high levels of egr1 and dusp1 expression. The anatomical drawings below the image show brain regions activated by hearing (medial brain section) or other conditions (lateral brain section), with vocal areas highlighted in red. White, gene expression, mRNA signal. Red, cresyl violet stain. Sections are sagittal. Scale bar  = 2 mm. (**I**) Quantification of dusp1 and egr1 expression. Values significantly above 1 indicate induced expression in singing animals (n  = 4, except for DLM and Uva n  = 3) relative to average of silent controls (n  = 3). Birds that sang >83.0 sec (>34 song bouts) in 30 min were used. The standard deviations of expression were large due to differences in singing amount (see **Fig. 4A**). Overall differences were significant (p<0.001, repeated measure ANOVA between singing and silent groups). * p<0.05, ** p<0.01, and *** p<0.001, unpaired *t*-test in each nucleus relative to silent control. Error bars, ±SD. The highest to lowest levels for dusp1 were in NIf, LMAN, Uva > HVC > DM, DLM, X, RA (p<0.01, ANOVA); For egr1 - AreaX > HVC > LMAN > NIf > RA > Uva, DLM (p<0.05, ANOVA). Abbreviations: A, Arcopallium; aIH, anterior part of the intercalated layer of the hyperpallium; H, hyperpallium; Hp, hippocampus; M, mesopallium; MD, dorsal mesopallium; MV, ventral mesopallium; N, nidopallium; Rt, nucleus rotundus; St, striatum; v, ventricle. For other anatomical abbreviations, see Table 1.

In songbirds, egr1 mRNA expression is robustly up-regulated in higher order sensory neurons by sensory stimuli, in movement-activated areas by limb and body movements, and in all telencephalic song nuclei by singing. However, egr1 is not up-regulated in the sensory input areas by sensory stimuli, including in the Entopallium (E, visual), Basorostralis (B, somatosensory), and L2 (auditory) (**Fig. 2A–C**; [29], [38]–[41]). Dusp1 expression shows the opposite pattern; it is not up-regulated in higher order sensory neurons by sensory stimuli, nor in movement-activated areas by movement, but is up-regulated in the sensory input areas E, B, and L2 by sensory stimuli (**Fig. 2E–G**; [34]). Here we tested whether dusp1 was up-regulated by singing in the song nuclei of the zebra finch (*Taeniopygia guttata*), a songbird.

**Figure 3:**
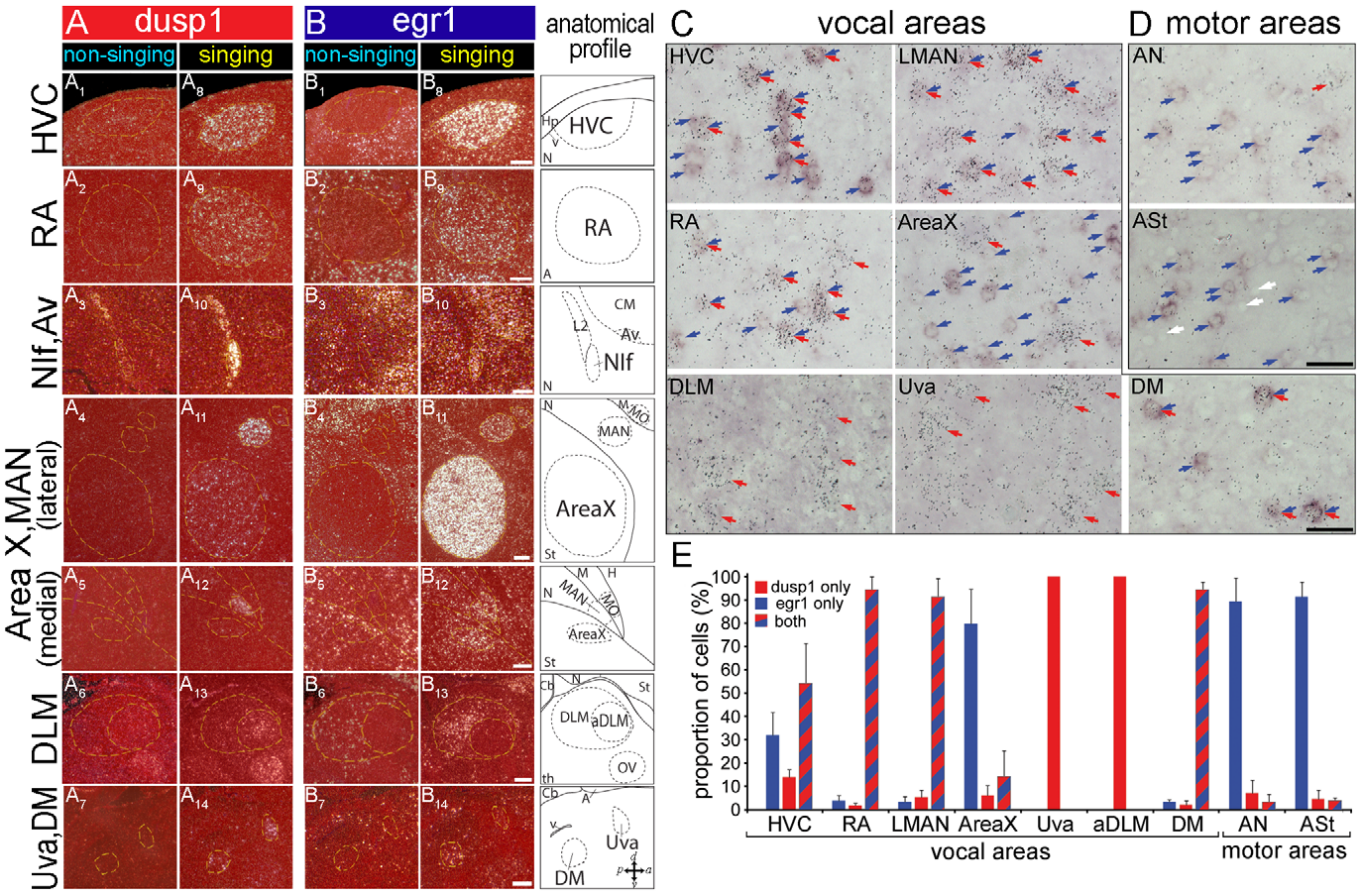
Magnified images of co-expressed dusp1 and egr1 mRNA in vocal areas and adjacent non-vocal areas. (**A**) dusp1 mRNA expression in song nuclei in a non-singing (**A_1–7_**), and singing (**A_8–14_**) male that sang for 30 min. (**B**) egr1 mRNA expression in adjacent sections. Yellow dashed lines, Nissl-stained boundary of areas labeled in anatomical profiles in the right most column. Sections are sagittal; anterior is right, dorsal is up. Scale bars  = 200 µm. (**C**) Double-labeled images of vocal areas. Egr1 mRNA is labeled with DIG probe as a purple/brown precipitate and dusp1 mRNA is labeled with a S^35^-probe detected by silver grains. Colored arrows refer to single dusp1 (red), single egr1 (blue), and double labeled (red/blue) cells. (**D**) Double-labeled images of movement-activated areas adjacent to LMAN (AN) and LAreaX (ASt). White arrows refer to examples of chromogenic background signals with a shadow effect (lighter inside the nucleus), which we used to locate individual cells. Orientation: Dorsal is up and anterior to the right. Scale bars  = 20 µm. (**E**) Proportion of single and double labeled cells in each area. The relative distribution of double-labeled cells among vocal areas and motor areas are significantly different (p<0.05 and <0.001; AreaX vs ASt and LMAN vs AN, respectively; n  = 3 animals; ANOVA). The distribution of labeled categories in RA and LMAN are significantly different from Area X (p<0.05, ANOVA), where in the latter only large cells are dusp1-labeled and small cells are either egr1-labeled or double-labeled.

In stark contrast to our expectation, we found that singing caused robust up-regulation of dusp1 in most zebra finch telencephalic song nuclei (**Fig. 2H,I**; higher magnifications in **Fig. 3A**). These nuclei included the following: HVC (a letter based name) of the nidopallium, the robust nucleus of the arcopallium (RA), and the interfacial nucleus of the nidopallium (NIf), in the posterior song pathway; and the lateral and medial portions of the magnocellular nucleus of the nidopallium (LMAN, MMAN) and the lateral portion of striatal nucleus Area X (LArea X), in the anterior song pathway (**Figs. 2H** **and** **3A**). Dusp1 expression was not induced in the medial portion of Area X (MArea X) or the oval nucleus of the mesopallium (MO. Only a few isolated cells were labeled in avalanche (Av) of the mesopallium (**Figs. 2H** **and** **3A**). To be certain that egr1 was induced in these same animals, we hybridized adjacent sections with a probe for egr1 mRNA and found robust singing-induced egr1 expression in all song nuclei, including in MArea X and MO (**Figs. 2D,I** **;** higher magnification in **Fig. 3B**).

In the brainstem, consistent with past findings [39], [40], egr1 mRNA was robustly up-regulated in the midbrain dorsal medial (DM) vocal nucleus of singing animals, but not in the thalamic song nucleus Uvaeformis (Uva) or in the dorsal lateral nucleus of the medial thalamus (DLM; **Figs. 2D,I** **and** **3B**; although a past study mentioned slight egr1 up-regulation in Uva [40]). Dusp1 was also up-regulated in DM. In contrast to egr1, we also found robust up-regulation of dusp1 in Uva and in a restricted anterior nucleus within DLM (aDLM; **Figs. 2H,I** **and** **3A**; originally described in Wada et al 2004 [**42**]). The latter appeared to be in the same part of DLM that receives a projection from Area X and projects to LMAN (according to the images in [43], [44]). Singing-related up-regulation of dusp1 expression was not visible in other parts of DLM.

Although dusp1 and egr1 were both expressed in the same song nuclei, the relative expression levels differed (**Fig. 2I**). Dusp1 was highest in NIf, LMAN, and Uva, and it was lowest in Area X (**Fig. 2I** red bars). Egr1 was highest in AreaX and lowest in LMAN (**Fig. 2I** blue bars). These findings indicate that dusp1 and egr1 expression occur in the same regions within the telencephalic song nuclei and DM, unlike the rest of the brain.

### Dusp1 and Egr1 are Co-expressed in Song Nuclei Neurons

We wondered whether the high induction of dusp1 and egr1 in the same song nuclei and DM was due to neurons with high levels of dusp1 being intermingled with other neurons that had high levels of egr1. Another possibility is that both genes were expressed in the same cells. To test for these alternatives, we performed double-labeling for dusp1 and egr1 mRNAs on brain sections from animals that sang for 30 min. In contrast to the rest of the brain, we found that the vast majority (>95%) of cells labeled in RA, LMAN, and DM were double-labeled for dusp1 and egr1 (**Fig. 3C,E**). HVC also contained a large proportion of double labeled cells (55%). The relationship was reversed for the striatal song nucleus Area X, where most of the labeled cells (∼85%) only expressed either egr1 or dusp1 (**Fig. 3C,E**). Within Area X, cells positive only for egr1 appeared to be smaller, characteristic of the medium spiny neurons of the striatum (**Fig. 3C**; [45]); cells positive only for dusp1 appeared to be larger and sparsely distributed, characteristic of the pallidal-like neurons of the striatum [46], [47]; cells positive for both dusp1 and egr1 (∼15%) were of the small type (**Fig. 3C**). In the thalamic song nuclei Uva and aDLM, we found only cells positive for dusp1 (**Fig. 3C,E**).

In these same animals, there was increased egr1 expression in some of the areas adjacent to the song nuclei (**Fig. 2D**). This expression was presumably due to the movements and sounds that the animals made while singing [29]. In these areas, such as the anterior striatum (ASt) caudal-ventral to Area X and the anterior nidopallium (AN) caudal to LMAN, the vast majority (∼92%) of labeled cells expressed only egr1 (**Fig. 3D,E**). There were some cells positive for only dusp1 (<6%), and cells co-expressing dusp1 and egr1 were rare (<2%; **Fig. 3E**). We did not find any large cells expressing dusp1 in ASt, unlike in Area X. The differences in distributions between ASt and Area X, and between AN and LMAN, were significant (p<0.05, p<0.001, respectively, ANOVA).

### Dusp1 Expression in Song Nuclei is Motor-driven

**Figure 4:**
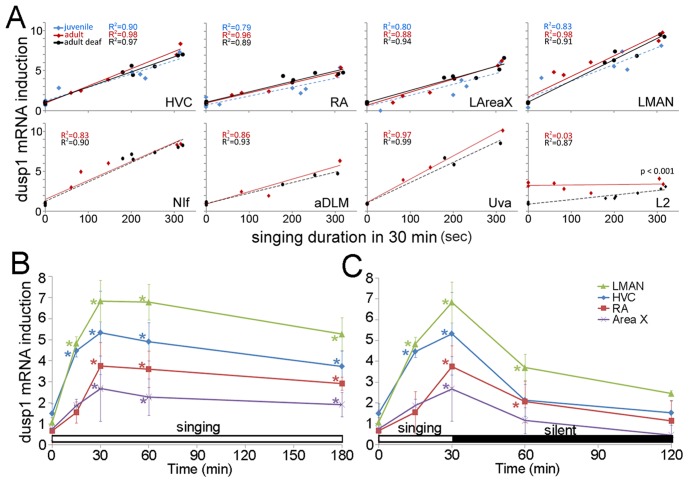
Temporal dynamics and auditory-feedback independence of singing-induced dusp1 expression. (**A**) Expression of dusp1 mRNA in intact adult (n  = 5), deafened adult (n  = 6), and juvenile subsong (n  = 5) singers in seven song nuclei (HVC, RA, NIf, LMAN, LAreaX, aDLM and Uva) and an auditory area (L2). Values were normalized by the average value in the same area of silent control animals of each group (n  = 3 each). Due to their small size, fewer singing samples were located for Uva (n  = 3 each group) and aDLM (n  = 3 each group). Lines represent the best fit of the data analyzed by simple regression (R^2^ and p-values, upper left). Only L2 showed a difference in intact and deaf animals (p<value, lower right, multiple regression). (**B**) Time course of continuous singing-induced dusp1 expression in birds that sang for various times, normalized to the average of silent controls (0 min). (**C**) Time course of discontinuous acute singing, where singing was stopped at 30 min. There was an overall difference among time points (p<0.001 in B and p<0.001 in C, repeated measures ANOVA), *p<0.05, Dunnett’s post test of each singing time point relative to silent controls (0 min). Values are averages ±SD.

We wondered if dusp1 expression in the song nuclei was motor-driven, similar to egr1 expression, or was sensorimotor-driven, *i.e.* required auditory feedback. This question was particularly relevant to dusp1 for two reasons: 1) dusp1 is robustly up-regulated by sensory stimuli only in sensory input neurons throughout the rest of the brain [34]; and 2) song system nuclei can show auditory responses (although mainly in anesthetized or sleeping zebra finches [48]–[50]). We found that dusp1 expression in song nuclei was not sensory-driven, as birds that heard 30 min of song playbacks without singing themselves did not show higher levels of dusp1 expression relative to silent controls (**Fig. 2E vs 2F**; overall difference: p  = 0.982, HVC: p  = 0.4661, RA: p  = 0.2318, NIf: p  = 0.0984, LMAN: p  = 0.1437, LAreaX: p  = 0.6281, aDLM: p  = 0.1007, Uva: p  = 0.3277, repeated measures ANOVA followed by unpaired *t*-test between silent [n  = 3] and hearing [n  = 3] groups). In contrast, in birds that sang different amounts of song for 30 min (those from **Fig. 2I**), like egr1 [39], the amount of dusp1 produced in song nuclei was linearly proportional to the amount of song produced (**Fig. 4A** **,** red diamonds and lines). In deafened birds, dusp1 expression was still induced in song nuclei by singing and was still linearly proportional to the amount of song produced. Moreover, this correlation in the deaf animals was no different than that observed in hearing-intact singing animals (**Fig. 4A** **,** black circles and lines; multiple regression analyses). A time course experiment showed that, like egr1 [29], [39], [51], dusp1 expression in song nuclei was acutely induced within 15 minutes of singing, peaked by 30 min, and was maintained thereafter as long as the birds continued to sing (**Fig. 4B**). When singing was interrupted at 30 min, dusp1 expression declined over the following 30 min and almost reached pre-singing baseline levels within 90 min (i.e. 120 min from the start of singing; **Fig. 4C**). The relative differences in dusp1 expression among song nuclei were maintained over time (**Fig. 4B**). These results demonstrate that unlike the sensory-driven dusp1 expression in sensory input neurons, dusp1 expression in the zebra finch song system does not require auditory input and is motor-driven like egr1 expression.

To assess whether singing-driven regulation of dusp1 mRNA expression is influenced by song plasticity, we investigated dusp1 expression in juveniles that produced subsong during a plastic stage of song development. We found no difference in the proportional level of dusp1 expression in song nuclei (**Fig. 4A** blue diamonds and dashed lines). These findings suggest that dusp1 expression in song nuclei is not influenced by the developmental differences between juvenile (plastic) and adult (crystallized) song.

Prior studies have shown that the zebra finch auditory area field L (L1, L2, and L3 not defined) displays neuronal firing preceding singing behavior, suggestive of some motor activity [52]. We tested for an influence of singing motor activity on dusp1 expression in L2. We found that the amount of dusp1 expression in L2 was not different between birds that did not sing but heard noises they made by moving around the cage, and birds that sang and heard these noises and songs of themselves (p  = 0.58; unpaired t-test between non-singing [n = 3] and singing [n = 5] groups). In these hearing intact birds, dusp1 expression in L2 did not correlate with the amount of song produced (**Fig. 4A**, L2 red line). In deaf birds that did not sing, as expected, dusp1 expression was lower in L2 relative to hearing-intact non-singing controls. However, in the deaf birds that sang, dusp1 expression in L2 was slightly, but significantly increased, resulting in a linear correlation with the amount of song produced (**Fig. 4A**, L2 black line). The regression lines were significantly different between hearing-intact singers and deaf singers (**Fig. 4A**, L2 red vs black line, multiple regression). Although similar results have not been reported for egr1 in higher order auditory neurons [39], it is possible that such a small difference was previously missed. These findings suggest that hearing-induced dusp1 is dominant in L2 but there could be some motor-driven activity in L2 that is unmasked by deafening. This idea can be tested specifically for L2 in the future using electrophysiological recordings.

### Dusp1 is Not Induced at High Levels in Non-vocal Motor Systems by Strong Motor Activity or Even Seizures

**Figure 5:**
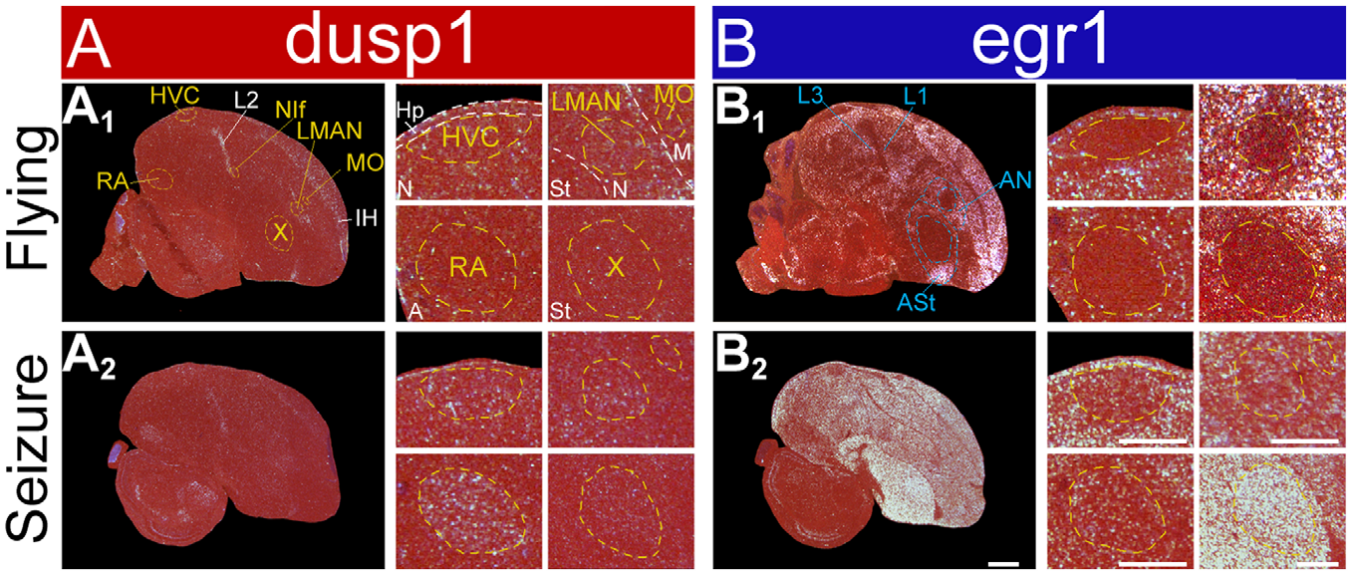
Lack of strong induction of dusp1 in areas adjacent to song nuclei. (**A**) Representative images of dusp1 expression in two groups of birds: **A_1_** flying, **A_2_** metrazole-induced seizure. White, gene expression, mRNA signal; red, cresyl violet cellular stain. (**B**) Adjacent sections hybridized with egr1. Scale bars  = 1 mm for whole brains, and 500 µm for high power images of song nuclei.

Even in over exposed *in-situ* images of singing animals, we found very low dusp1 expression and high egr1 expression in areas adjacent to song nuclei (**Fig. S1A,B**). We wondered if dusp1 and egr1 have different thresholds for induction in motor systems. We reasoned that 30 min of singing activity is enough to induce dusp1 expression in song nuclei, but 30 min of movement activity might not be enough to overcome the threshold in the adjacent regions [34]. To test this idea, we examined dusp1 expression in two groups of animals: 1) those that flew vigorously for 60 min while being chased in a room by an experimenter, and could also hear and see; and 2) those with seizures induced by the GABAergic antagonist metrazole or the excitatory glutamate receptor agonist kainate. The results from flying animals were consistent with prior findings; dusp1 was only up-regulated in primary sensory populations (e.g. L2 and IH), and egr1 was up-regulated in many higher order sensory (L1 and L3) and motor (ASt and AN) areas, including those adjacent to song nuclei (**Fig. 5A_1_,B_1_**; [29], [34]). In the seizure groups, egr1 was strongly up-regulated throughout the brain, except in sensory input neurons, as expected (**Figs. 5B_2_** **and S1D**; [29], [53]). Dusp1 was still only up regulated in sensory input areas (such as L2) and in pallial song nuclei (HVC, RA, and LMAN; **Figs. 5A_2_** **and S1C**). These findings support our conclusion that motor-driven regulation of dusp1 expression is specialized in song nuclei.

### Dusp1 is Regulated by Singing in the Song Nuclei of Other Vocal Learning Avian Lineages

We next wondered if specialized motor-driven regulation of dusp1 expression in song nuclei was also present in the two other vocal learning lineages: parrots and hummingbirds. Thus, we tested for dusp1 expression induced by singing in a parrot and several hummingbird species.

### Parrot

**Figure 6:**
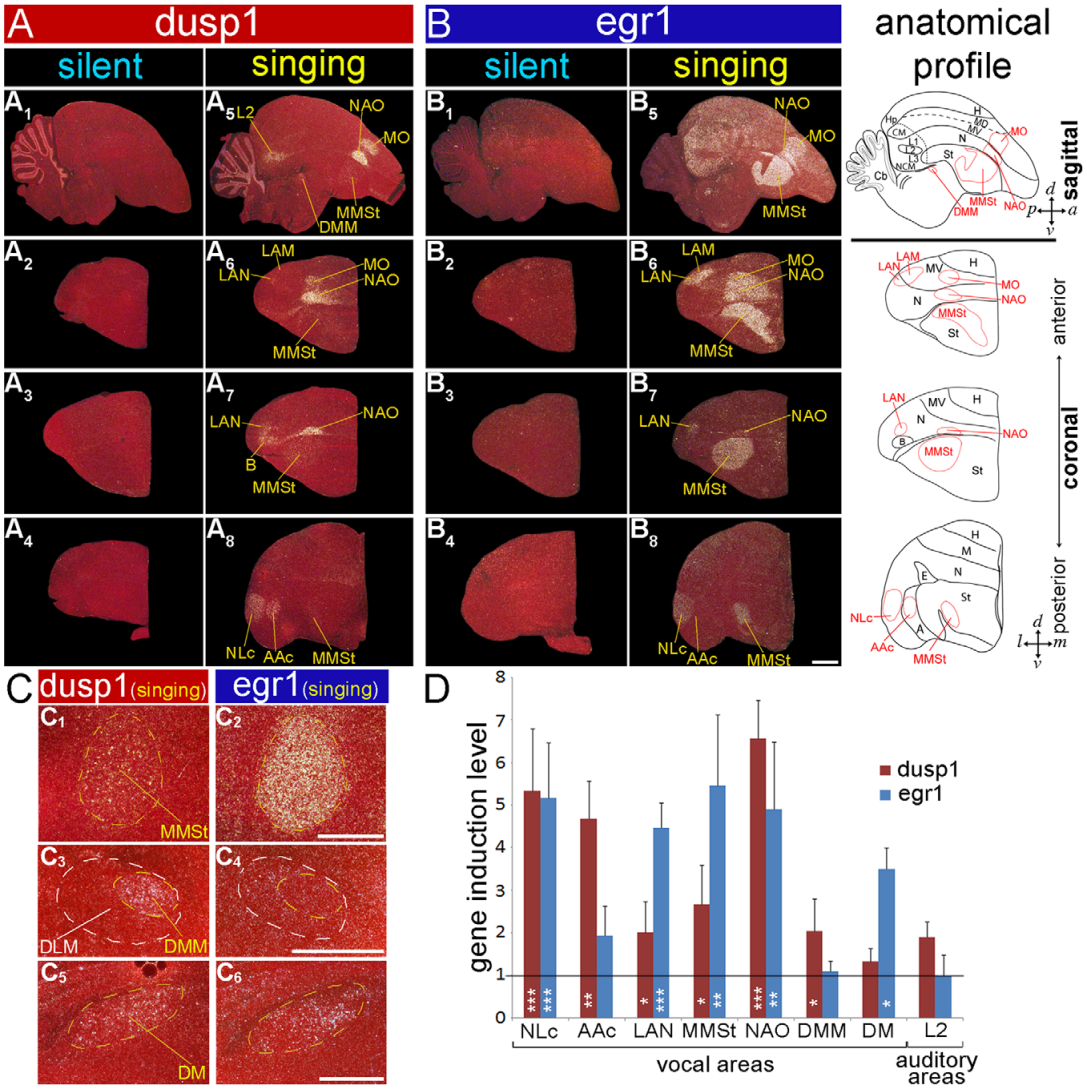
Dusp1 and egr1 mRNA expression in budgerigar brain after singing. (**A**) Darkfield images of *in situ* hybridization with dusp1 from a non-singing control (**A_1–4_;** no auditory stimulus, sitting relatively still) and a singing (**A_5–8_**) male bird that produced warble song for 30 mins. (**B**) Adjacent sections hybridized with egr1. Sagittal (**A_1,5_**, **B_1,5_**) and coronal (**A_2–4,6–8_**,**B_2–4,6–8_**) sections are shown. The right most column shows anatomical profiles with vocal areas highlighted in red; only the core of the MO and NAO song nuclei where we observe the dusp1 expression is drawn. (**C**) Magnified images of dusp1 and egr1 mRNA expression in the nuclei indicated after singing. (**D**) Quantification of dusp1 and egr1 expression. Values significantly above 1 indicate induced expression in singing animals (n  = 3) relative to the average of silent controls (n  = 3, overall difference p<0.001 repeated measures ANOVA; * p<0.05, ** p<0.01, and *** p<0.001 unpaired t-test for each brain region relative to silent controls). Error bars, ±SD. Scale bar  = 2 mm in B_8_ (applies to all A and B); 1 mm in C_2_ (applies to C_1,2_), C_4_ (applies to C_3,4_), and C_6_ (applies to C_5,6_).

We found that six of the seven telencephalic song nuclei in the budgerigar (*Melopsittacus undulates*) showed robust singing-induced expression of dusp1: MO (analog of songbird MO), NAO (MAN analog), and MMSt (Area X analog) of the anterior vocal pathway; and NLC (HVC analog), AAC (RA analog), and LAN (NIf analog) of the posterior vocal pathway (**Figs. 1** **and** **6A,C,D**). The only nucleus with no visible dusp1 expression was LAM (analog of songbird Av; **Fig. 6A** **_2_,A_6_**). The highest dusp1 expression occurred in NAO (LMAN analog), NLC (HVC analog), and AAC (RA analog), and the lowest occurred in the striatal song nucleus MMSt (Area X analog). This expression pattern was not identical, but was similar to the pattern observed in the zebra finch song nuclei. Likewise, the expression in budgerigar MMSt was sparse and appeared to be highest in the larger neurons (**Figs. 6C_1_** and **S2**). Egr1 was up-regulated in all seven telencephalic song nuclei of these same animals (**Fig. 6B,C,D**; expression in AAC was low in the animal shown). In the thalamus, there was robust singing-induced expression of dusp1 but not egr1 in DMM within DLM (analog of songbird aDLM; **Fig. 6C** **_3,4_**). In budgerigars that heard playbacks of warble song (**Fig. S3A,B**; [34]) or heard themselves sing (**Fig. 6A,B**), there was induced dusp1 expression in the auditory input neurons of the telencephalon (L2) and induced egr1 expression in the adjacent secondary (NCM) and tertiary (CM) auditory neurons. Thus, the dusp1 and egr1 expression patterns in the auditory areas were complementary. The budgerigar song playbacks did not induce dusp1 (or egr1, as expected [54]) in the budgerigar song nuclei (**Fig. S3A,B**; [**34**]).

There were some differences in budgerigars relative to zebra finches. In budgerigars, a part of nucleus basorostralis (B, primary somatosensory neurons) ventral to LAN showed dusp1 expression in the singing animals (**Fig. 6A** **_7_**); this part of B is where the somatosensory representation of the beak is located in budgerigars [55]. In contrast, dusp1 expression in B of singing zebra finches was not as strong. Expression of dusp1 in the budgerigar MO (MO analog) and NAO (LMAN analog) was highest in the cores of these nuclei. By contrast, egr1 in budgerigars and both genes in zebra finches were expressed evenly throughout each of the song nuclei. Budgerigars are thought to lack a nucleus equivalent to songbird Uva [56], and we did not find singing-induced dusp1 expression in any thalamic nucleus other than DMM. We noted some expression of both dusp1 and egr1 in the parrot midbrain nucleus DM (**Fig. 6C** **_5,6_**), but expression levels were lower than in the zebra finch. Despite these differences, the findings suggest that dusp1 induction in song nuclei of the budgerigar is motor-driven, as it is in the zebra finch.

### Hummingbirds

**Figure 7:**
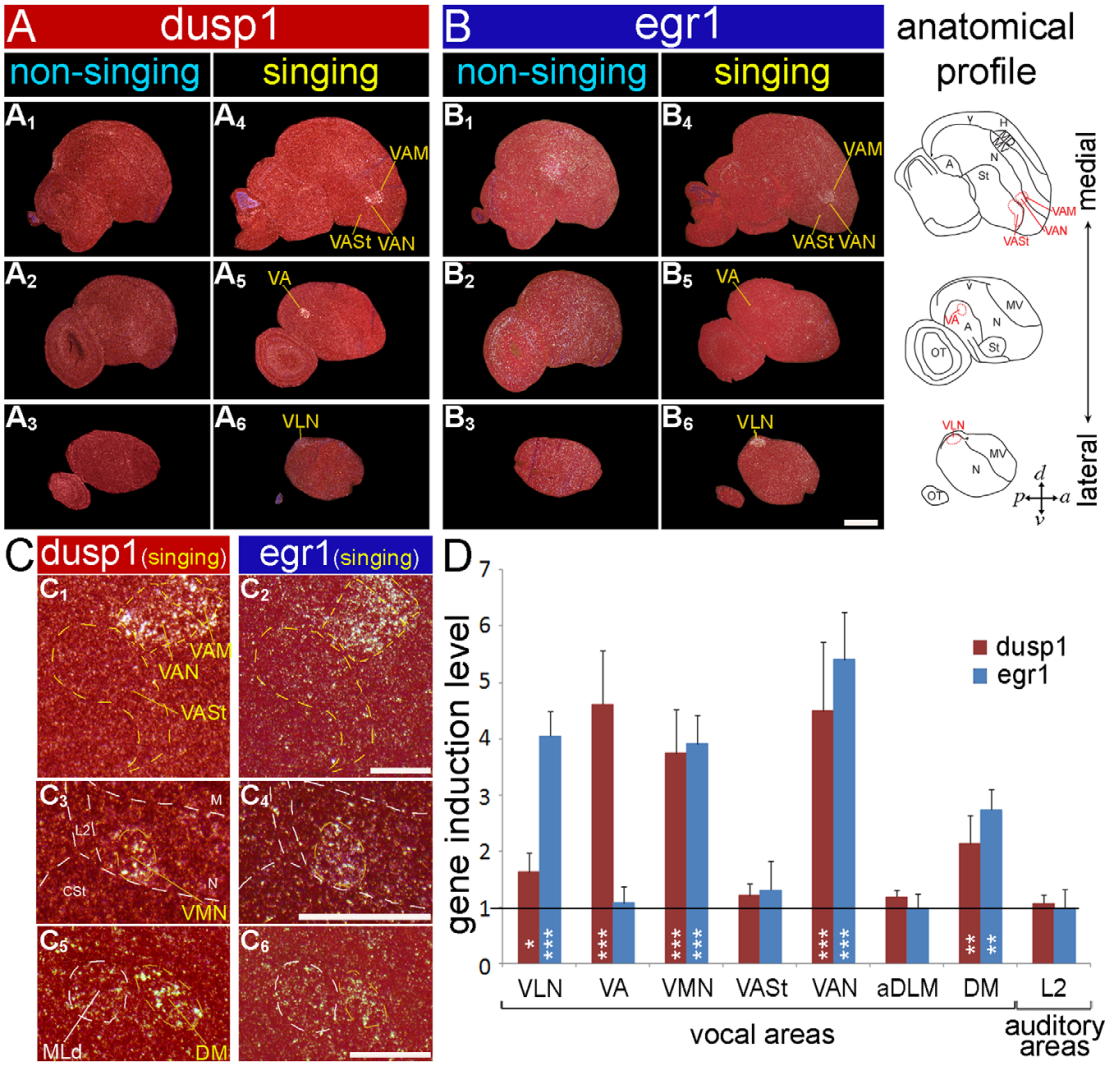
Dusp1 and egr1 mRNA expression in sombre hummingbird brain after singing. (**A**) Darkfield images of medial to lateral sagittal sections hybridized with dusp1 from a non-singing control (**A_1–3_;** no auditory stimulus, but flying) and a singing sombre hummingbird (**A_4–6_)** that sang for 30 min. (**B**) Adjacent sections hybridized with egr1. The level of egr1 induction in VA and VASt of the singing animal shown is low. White, gene expression, mRNA signal; red, cresyl violet stain. The right most column shows anatomical profiles with vocal areas highlighted in red. (**C**) Magnified images of dusp1 and egr1 mRNA expression in several song nuclei and in DM after singing. (**D**) Quantification of dusp1 and egr1 expression in vocal areas and in L2 after singing. Values significantly above 1 indicate induced expression in singing animals (n  = 3) relative to average of silent controls (n  = 3, overall difference p<0.001 repeated measures ANOVA; * p<0.05, ** p<0.01, and *** p<0.001 unpaired *t*-test for each brain region relative to silent controls). Error bars, ±SD. Egr1 and/or dusp1 induction in VA, VASt, aDLM was only expressed in animals that sang the most, and thus an overall significant difference is not seen when averaging across the animals used. Scale bar  = 1 mm in B_6_ (applies to all A and B); 500 µm in C_2_ (applies to C_1,2_), C_4_ (applies to C_3,4_), and C_6_ (applies to C_5,6_).

There are two main lineages of hummingbirds, the hermits (*Phaethorninae*) and non-hermits (*Trochilinae*) [57], [58]. We found that five of the seven telencephalic song nuclei showed singing-induced dusp1 expression in the sombre hummingbird (*Aphantochroa cirrochloris*), a non-hermit: VLN (analog of songbird HVC), VA (RA analog), VAN (MAN analog), VAM (MO analog), and VMN (NIf analog; **Figs. 1** **and** **7A,C,D**). The strongest dusp1 expression occurred in VAN (MAN analog) and VA (RA analog), and minimal to no induced expression in the striatal song nucleus VASt (Area X analog; **Fig. 7C_1_,D**). This pattern was not identical, but similar to the patterns observed in the zebra finch and budgerigar. In the Anna’s hummingbird (n = 2 singing; 1 silent control), another non-hermit, the pattern of singing-induced dusp1 expression was nearly identical to the sombre hummingbird (**Fig. S4A_1,2_ vs S4B_1,2_**). In the rufous-breasted hermit (n = 2 singing; 1 silent control), the expression pattern was reversed, with higher dusp1 expression in VLN than in VA (**Fig. S4C_1,2_**). Egr1 was strongly expressed in all pallial telencephalic song nuclei of these same animals (**Fig. 7B,C,D**) [22]; however, in VA and in the striatal song nucleus VASt, egr1 expression is robust only in animals that sang most, resulting in no significant difference in egr1 expression between singing and silent animals (**Fig. 7D**). The observed species differences could represent a difference between species’ lineages or simply individual variation; resolving this ambiguity will require sampling more animals of different species. No singing-induced dusp1 (or egr1 as expected; [22]) expression was detected in the song nuclei of sombre hummingbirds that heard song playbacks (n = 3; data not shown). Some of the hummingbirds, which were flying before brain dissection, shows induction of egr1 expression in brain areas adjacent to song nuclei (**Fig. 7B** **_1–3_**; [22], [29]); however, we did not observe strong dusp1 expression in the same areas (**Fig. 7A** **_1–3_**). We also noted singing-induced dusp1 expression (but no egr1 expression) in a thalamic nucleus similar to aDLM, located dorsally in DLM of hummingbirds relative to songbirds and parrots (**Fig. S4A_3_,B_3_,C_3_**). There was notable singing-induced dusp1 and egr1 expression in the midbrain nucleus DM of hummingbirds (**Fig. 7C** _5,6_,D). We did not find a nucleus similar to Uva. These findings show that hummingbirds exhibit singing-induced dusp1 expression in song nuclei that appears to be motor-driven, as it is in the songbird and parrot.

### Dusp1 is Not Regulated by Singing in the Forebrain of Vocal Non-learning Avian Lineages

The specialized dusp1 expression in song nuclei of multiple vocal learning lineages raises the question of whether dusp1 expression is also induced by singing in the forebrain of vocal non-learning species. Although prior studies have claimed that vocal non-learners lack forebrain song nuclei, (reviewed in [11]), this claim had never been tested using activity-dependent gene expression. Here we assayed for singing-driven dusp1 expression in two species that have been demonstrated to be vocal non-learners [28], [59]: the Eastern phoebe (*Sayornis phoebe*), a suboscine bird and close relative of songbirds; and the ringdove (*Streptopelia risoria*), of the order Columbiformes and considered a member of a close sister group to the group that includes hummingbirds (**Fig. 1**). The male Eastern phoebe exhibits singing behavior that is used to attract mates, and the male ring dove has a song-like cooing behavior that is used for courtship and territorial defense; the acoustic structure of the songs/coos is innate in both species [28], [59]. After males produced these vocal behaviors in similar amounts as the vocal learners, we examined dusp1 expression in serial sections throughout the brain relative to silent controls.

**Figure 8:**
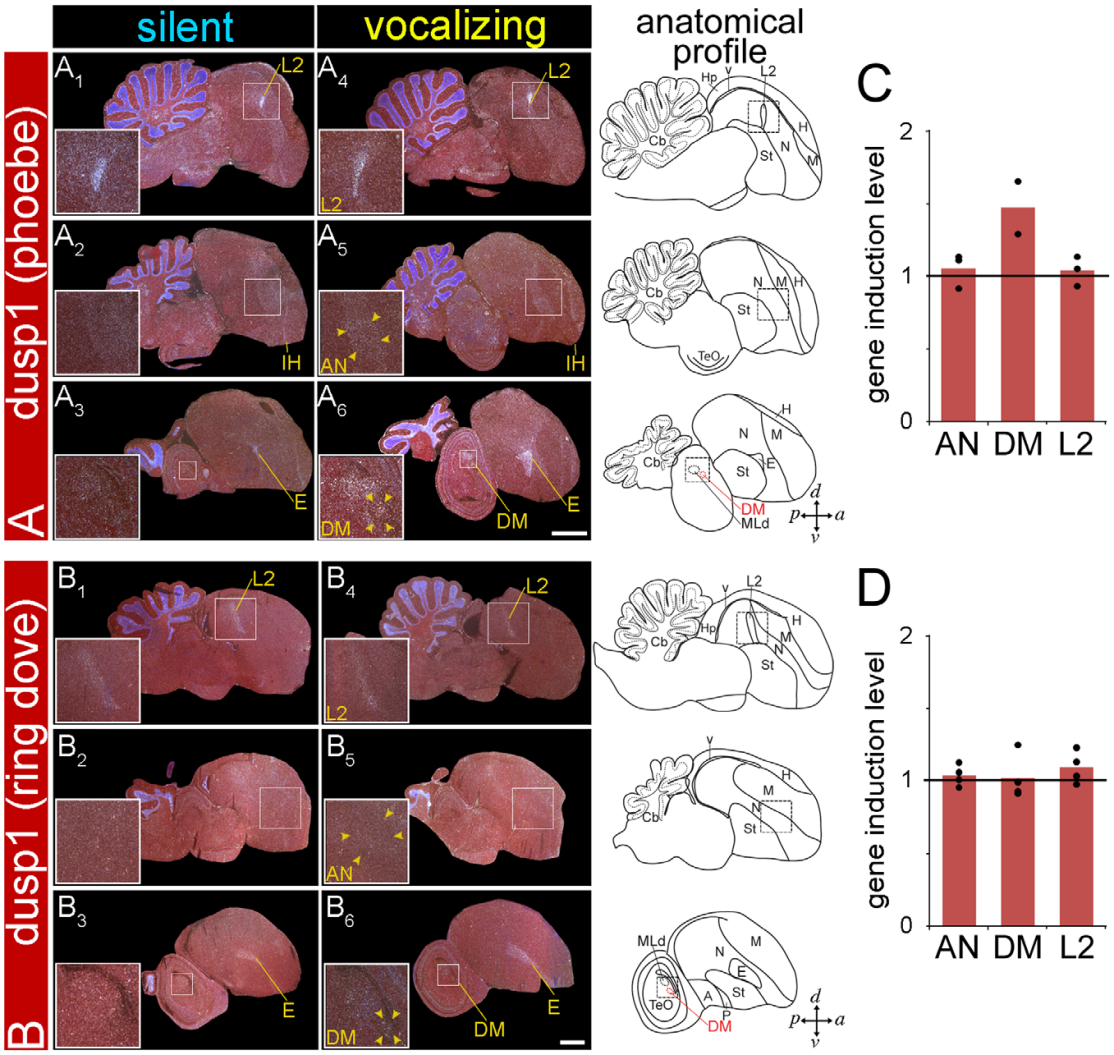
Dusp1 mRNA expression in the brains of vocal non-learners after singing. Darkfield images of *in situ* hybridizations from medial to lateral sagittal series with dusp1 from Eastern phoebes (**A**) and ring doves (**B**). Shown are brain images from silent control male birds (**A_1–3_,** **B_1–3_**; no auditory stimulus) in a sound attenuation chamber and male birds that sang (phoebe) or cooed (ring dove) for 30 minutes (**A_4–6_**, **B_4–6_**). Inset shows areas highlighted in boxes and quantified: L2, AN, and DM. White, gene expression, mRNA signal. Red, cresyl violet stain. Lines and names in yellow, areas where each mRNA was robustly induced. Anatomical profiles to the right show vocal brain areas (DM) highlighted in red and non-vocal areas in black. Scale bars  = 2 mm. (**C**) Quantification of dusp1 expression in phoebes. (**D**) Quantification of dusp1 expression in ring doves. Values significantly above 1 indicate induced expression in vocalizing animals (n  = 3 for AN and L2; n  = 2 for DM of phoebes, n  = 4 for ring doves) relative to the average of silent controls (n  = 4 for phoebes, n  = 3 ring doves; un-paired *t*-test). No significant difference was found.

We found no evidence of singing-induced dusp1 expression in the telencephala of Eastern phoebes and ring doves (**Fig. 8A,B**). There was high expression in the sensory input neural populations (i.e. auditory L2; visual E; somatosensory B), as expected [34]. The latter findings confirm that the zebra finch dusp1 probe worked in these animals as it did in parrots and hummingbirds in the present study, and other species in prior studies [34]. We could not find even a small quantitative increase in dusp1 expression within AN (**Fig. 8C,D**), the region where we would expect to find the analog of LMAN, the nucleus in vocal learners with the highest dusp1 expression. We did not detect a thalamic area with greater dusp1 expression after singing that would be equivalent to aDLM. In contrast, the midbrain vocal nucleus DM did show dusp1 expression in the Eastern phoebes that sang (**Fig. 8A,C**) and in the ring dove that cooed the most (**Fig. 8B,D**); DM is present in all avian species, and it is necessary for and active in the production of innate vocal patterns [**31**]. Even in the animal that cooed the most, there were no forebrain areas with cooing-induced dusp1 expression. Thus, we do not believe that the amount of vocalizing can explain the differences between vocal learners and non-learners. These findings show that the vocal non-learning species tested thus far do not have forebrain regions with specialized singing-driven dusp1 expression.

### The Upstream Genomic Sequence of Dusp1 in Avian Vocal Learners and Non-learners

**Figure 9:**
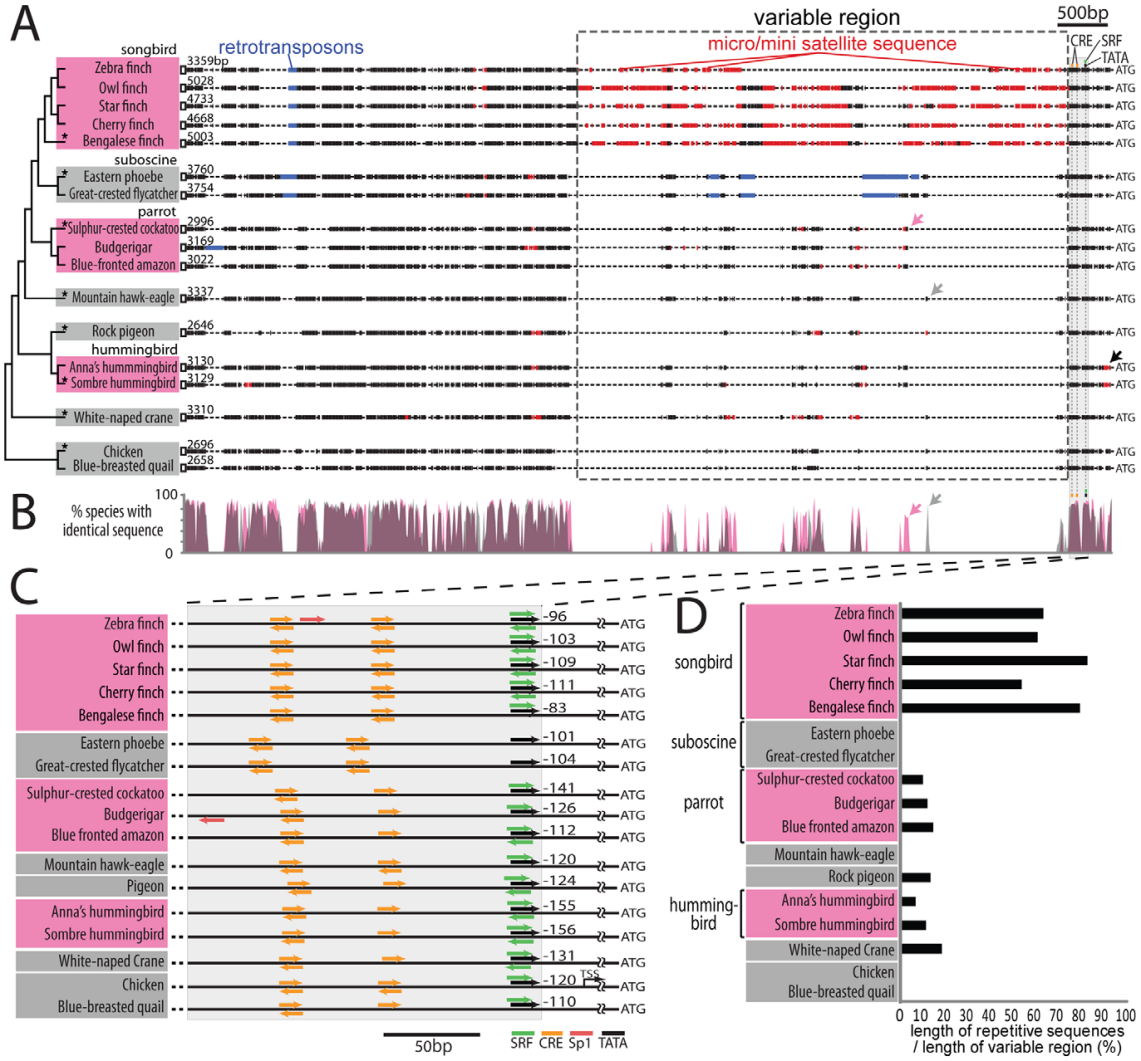
Upstream sequences of dusp1 among species. (**A**) Schematic of the ∼3 kb upstream region of dusp1 in vocal learning and vocal non-learning avian species (range 2,646 to 5,048 bp depending on species). The conserved region used to clone the sequence among the avian species is indicated by an open box at the 5′ end. ATG is the initiation codon of the protein. Red boxes, repetitive microsatellite sequences. Blue boxes, retrotransposon sequences (MIR3/LINE-like and CR1/SINE-like elements in the songbirds, suboscines, and one parrot species). Arrows indicate the similar sequences found only in vocal learners (pink) or in vocal non-learners (grey). The black arrow indicates microsatellite sequence close to the start codon found in hummingbirds. Grey shaded region, proximal regulatory region where the putative *cis*-binding sites are found. (**B**) % of species with identical sequences among at least 2–3 vocal learners (pink) or 3 or more vocal non-learner lineages (grey). Overlap is pinkish-grey. (**C**) Region within 300 bp of the transcription start site (ATG), showing putative *cis*-binding sites for activity-dependent transcription factors (color-coded for individual transcription factors); direction of arrows indicates strand on which the binding motif was found (forward + strand; backward - strand). Translation start site (TSS) annotated in chicken genome. (**D**) Proportion of repetitive microsatellite sequence in the variable region between species (dashed boxed region in (A)).

The above findings indicate that there might be something different about the regulatory regions of the dusp1 gene in vocal learners that result in specialized motor-driven expression in song nuclei. We examined the sequence upstream to the coding region of dusp1 in the published chicken (*Gallus gallus*) and zebra finch genomes [60], [61]. However, we found that the zebra finch sequence was incorrectly assembled, apparently due to repetitive DNA sequences. Thus, we designed degenerate primers specific for two regions conserved between chicken and zebra finch: a region approximately 3 kb upstream of the dusp1 coding sequence and the beginning of the coding sequence. These primers were used in PCR reactions to clone the dusp1 upstream region from genomic DNA of zebra finch, chicken, and 15 other avian species (**Fig. 9A**). Sequence alignments (range from 2,646 to 5,048 bp depending on species) using Dialign [62] followed by maximum likelihood analyses [63] inferred a phylogeny that was similar to the one reported by Hackett et al. 2008 [14] (**Fig. 1** **vs** **Fig. 9A**). The inferred phylogeny included a distant relationship of hummingbirds to both parrots and songbirds. Transcription factor motif searches using the Cluster Buster program revealed the presence of multiple conserved sites within 300 bp upstream of the start codon in all species tested: a putative TATA box transcription site; a putative activity-dependent transcription factor binding site for the cAMP response element (CRE); and a putative activity-dependent transcription factor binding site for the serum response factor (SRF; **Fig. 9C**). These putative activity-dependent sites are consistent with dusp1’s response to neural activity [34], [64]. Although we noted minor differences between species in these putative activity-dependent sites and the putative TATA box, the differences did not segregate vocal learners and non-learners (**Fig. 9C**). In contrast, the groups did differ in the presence of microsatellite DNA. The largest microsatellite region occurred approximately 10 bp upstream of the 5' most CRE site, and ranged in size from 578 to 2817 bp, depending on the species. There were some specific sequences found either only in vocal learners or non-learners (**Fig. 9B** pink and grey arrows respectively). In addition, there was a tendency for vocal learners to have longer (**Fig. 9A** variable region) and/or more copies (for hummingbirds) of microsatellite repeats relative to their close vocal non-learning relatives (songbirds vs suboscine birds; parrots vs suboscine birds or eagle; **Fig. 9D**). In hummingbirds the microsatellite repeats were 3′ to the CRE sites and near the start codon. In summary, there was a correlation between the specialized motor-driven regulation of dusp1 expression in song nuclei and the presence of microsatellite sequences upstream of the dusp1 gene of vocal learners. Future investigations are necessary to determine whether these sequence differences are responsible for specialized dusp1 expression in vocal learners.

## Discussion

**Figure 10:**
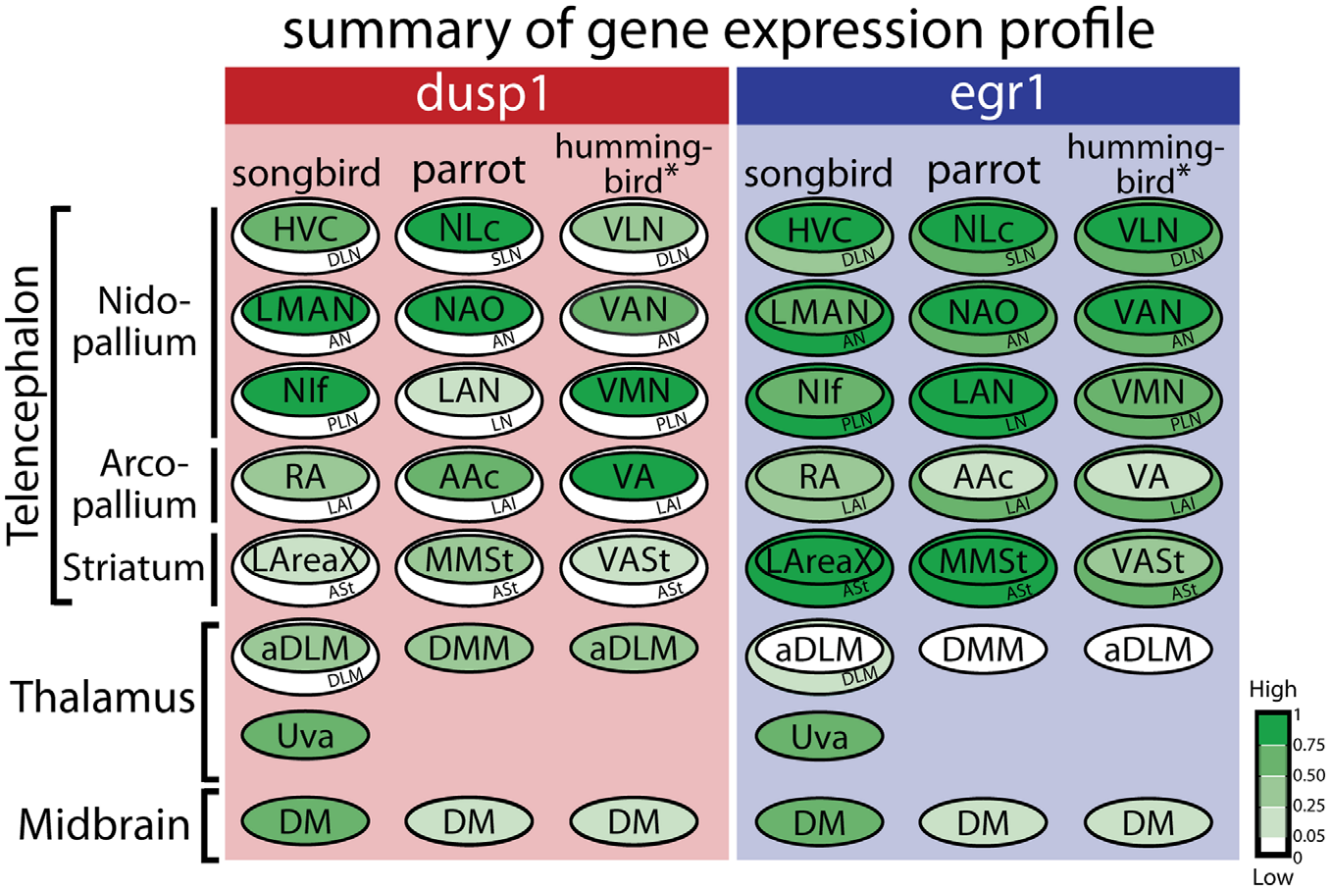
Summary of gene induction in vocal and movement-activated areas of vocal learners. Intensity of green indicates relative levels of activity-induced dusp1 or egr1 induction in each area, determined from the *in situ* hybridizations (see methods). White (0), no detectable induction; Dark Green (1), highest induction levels. Gene induction in song nuclei is due to singing, and in regions adjacent to song nuclei is due to moving. * The values for hummingbirds are an average of several species: sombre hummingbird (n  = 3 singing, n  = 3 silent), Anna’s hummingbird (n  = 2 singing, n  = 1 silent), and rufous-breasted hermit (n  = 2 singing, n  = 1 silent). For anatomical abbreviations, see Table 1.

Previous studies have shown that IEGs are expressed at similar levels in song nuclei after singing and the adjacent regions after performing other behaviors [29], [65]–[68]. We found that dusp1 was an exception to this pattern. Dusp1 showed strong motor-driven (singing-induced) expression in the song nuclei, but not motor-driven (hopping- or flying-induced) or sensory-driven (hearing-induced) expression in the areas adjacent to song nuclei (**Fig. 10A**). Moreover, dusp1 was co-expressed with egr1 in the same neurons within song nuclei (demonstrated in zebra finches), which does not occur in other brain areas. This specialized regulation in song nuclei occurred in all three avian vocal learning lineages tested but not in the forebrains of closely related vocal non-learning lineages. These findings suggest a specialized induction mechanism in song nuclei of vocal learners. If the phylogenetic evidence continues to support multiple independent origins of vocal learning, then this change in dusp1 regulation may have occurred independently multiple times. Below we discuss the implications of these findings.

### Convergent Regulation of Specialized Dusp1 Expression in Forebrain Song Nuclei

The motor-driven expression of dusp1 in song nuclei of vocal learners might reflect a convergent trait. Vocal non-learning avian species, including the Eastern phoebe and doves, reportedly lack forebrain song nuclei based on Nissl staining, the effects of lesions, tract tracing, and gene expression evidence [28]–[31], [42], [69], [70]. Our results of no activity-dependent dusp1 expression in the forebrain of singing animals support these prior conclusions. Alternative possibilities are that: 1) Vocal non-learners have rudimentary song nuclei that could have been missed even in serial sections throughout the brain due to being only a few neurons in size; or 2) They have non-specialized song nuclei, without dusp1 up-regulation. Nevertheless, all such possibilities lead to the same conclusion. There are clear differences in vocalizing-driven gene expression between vocal learners and non-learners examined thus far, suggesting independent evolution of motor-driven dusp1 expression in song nuclei of vocal learning birds and/or possible losses in suboscines and other species.

### Possible Functional Consequences of Specialized Dusp1 Induction

The activity-dependent expression of dusp1 in song nuclei of vocal learners and the conserved activity-dependent dusp1 expression in sensory input neural populations in birds and mammals suggest that song nuclei and sensory-input areas may share some properties. One possible shared property is high levels of activity, which could cause more cellular stress due to high calcium influx. Dusp1 is thought to be involved in protecting cells from physiological stress and subsequent programmed cell death by inactivating MAPK (ERK and JNK) and preventing the subsequent expression of IEGs (**Fig. S5A,C**; [36], [71]–[73]). The cellular stress hypothesis predicts that song nuclei and sensory input areas with high levels of dusp1 should have correspondingly high levels of metabolic activity. A number of previous discoveries support this hypothesis: 1) In songbirds, song nuclei and sensory input areas have the highest levels of cytochrome oxidase (a metabolism marker) in the telencephalon [74]. Changes in cytochrome oxidase activity within RA during song development positively correlate with the amount of spontaneous neural firing in RA [75]; 2) Our observation of the data in a recent study [76] suggests that both song nuclei and sensory input areas contain the highest levels of Perineuronal Net (PNN) labeled neurons in the telencephalon; PNN labeled neurons tend to have higher firing rates and PNN are thought to protect cells against oxidative stress [77]; 3) The firing rates in song nuclei and sensory input areas are higher than in the adjacent brain areas of both singing and silent zebra finches [45], [78]; 4) The act of singing causes up-regulation of many genes in songbird song nuclei, and many of the induced genes are involved in the cellular stress response and neuroprotection [68]; and 5) It was recently shown that parvalbumin (PV), known to buffer neurons from excess Ca^2+^ levels in neurons that fire at high rates, is enriched at higher levels in song nuclei of all three vocal learning avian lineages and in sensory input areas relative to other brain areas [79]. Based on the combined findings, we hypothesize the following functional role for dusp1 in the evolution of vocal learning: after the emergence of a basic vocal learning circuit, vocal learners gained specialized regulation of dusp1 (as well as PV and cytochrome oxidase) in the circuit to protect it from the higher levels of activity associated with the novel behavior. This hypothesis can be tested by determining whether knockdown of dusp1 increases the rate of cell death in song nuclei when birds sing.

### Potential Mechanisms of Specialized Motor-driven Dusp1 Expression in Song Nuclei

A mechanism of convergent, specialized motor-driven dusp1 expression in song nuclei would have to explain differential regulation in multiple song nuclei of each vocal learning avian lineage. One possible explanation is that there were convergent changes in the dusp1 regulatory region that affected its transcription only in song nuclei. Our analysis of the putative regulatory sequence upstream of the dusp1 gene mainly found differences in microsatellite repeats between vocal learners and vocal non-learners. Microsatellite insertions in promoters are known to produce rapid gene expression evolution by repositioning enhancers or suppressors farther or closer to the promoter [61], [80]. An alternative possibility is that an activity-dependent transcription factor capable recognizing and binding to a region in the dusp1 promoter was convergently expressed in song nuclei and in sensory input areas. Such a hypothesis has been demonstrated for other traits. Wing spots used for courtship displays evolved independently in various lineages of drosophila, and in each case mutations occurred in the *cis*-regulatory region of the gene that codes for the yellow pigment responsible for the spot color; the mutated *cis*-regulatory region is recognized by a specific homeobox transcription factor that is expressed in a spot pattern in all drosophila species [4]. In songbirds, there are two candidate genes with high expression in song nuclei and sensory input neurons in the absence of singing: calcium binding protein S100B, which is involved in neurite extension via microtubule assembly [81]; and cannabinoid receptor 1 (CB1) [82]. However, unlike dusp1, S100B most strongly expressed in HVC and throughout the midbrain, but not in LMAN. CB1 expression is equal in LMAN and Area X. Of the genes tested to date for differential expression in multiple vocal learning avian lineages, none have the same spatial pattern of expression as dusp1 [83]–[85]). The candidate gene that shows the most spatially correlated expression with dusp1 in zebra finches is another phosphatase (FAM40B); this gene shows high basal expression in sensory input areas of the thalamus and telencephalon, specialized expression in song nuclei, and is most strongly expressed in LMAN (Whitney, Pfenning, Howard, Blatti, Ward, Hartemink, Sinha, and Jarvis; submitted). FAM40B expression has not yet been examined in other vocal learning avian lineages.

To further explain our results, one would have to also postulate one or more modifications that partially dissociate dusp1 and egr1 regulation. Dusp1 has been shown to be a potent inhibitor of egr1 expression in neuroblastoma cells, where over-expression of dusp1 completely blocks stimulus-induced egr1 expression by inactivating ERK (known pathway illustrated in **Fig. S5A**; [35]). Our *in vivo* data from birds and mice are consistent with this mechanism (**Fig. S5A,B**; [34]). However, this mechanism cannot explain the co-expression of dusp1 and egr1 in song nuclei. In the song nuclei, dusp1-mediated down-regulation of egr1 would have to be dissociated, which could theoretically result from a mutation anywhere in the pathway, including dusp1 protein induction, ERK activation, and egr1 regulation (**Fig. S5C**). Such a mutation could be in the protein coding sequence of dusp1, in an interacting protein, or in dusp1 splice variants that are specific to the song nuclei and do not down-regulate egr1. All of the above hypotheses are testable.

### New Findings in the Thalamic Song Nucleus aDLM and the Auditory Area L2

Although our goal was to study dusp1 regulation in telencephalic song nuclei, two additional findings offer new insights into the functional organization of the song system and auditory areas. First, we were able to localize a thalamic nucleus that is activated by singing in all three vocal learning avian lineages: songbird aDLM; parrot DMM; and hummingbird aDLM. The exact boundary of DLM as a song nucleus in songbirds has not been inconsistently defined [43], [44], [46], [86], [87]. The original designation of DLM was based on pigeons, a vocal non-learner, and investigators labeled the entire dorsal thalamic area as DLM [88]–[90]. However, in budgerigar, a song nucleus DMM was clearly defined as being within DLM [19], [20], [54]. Molecular mapping of this nucleus using dusp1 for each vocal learning avian lineage unambiguously placed it ‘within’ the larger DLM nucleus. In support of our conclusion, a recent electrophysiology study in zebra finches found song premotor activity only in an anterior part of DLM [91]. The aDLM location we mapped matches the projection field from Area X and to LMAN [43], [44]. It was named anterior DLM (aDLM) in a previous study due to its differential Nissl staining and differential expression of 8 out of 20 glutamate receptors, similar to expression patterns in the telencephalic song nuclei [42]. Similar features were not found in the DLM of ring doves, where the expression of glutamate receptors was the similar as the areas surrounding zebra finch DLM [42]. Johnson et al 1995 [43] and Bottjer et al. [92] noted that DLM had a dorsolateral part that projects to LMAN, and a ventromedial part that projects to the nidopallium around LMAN. Based on calbindin expression and other evidence, Pinaud et al. [93] proposed calling these areas the DLM core and shell, respectively, and argued that the surrounding area is really a larger DLM structure. We believe that all of these different designations (aDLM, DLM core, and dorsolateral DLM) refer to the same area that is connected to the song system, which we prefer to call aDLM, as an anterior specialized part of DLM. The high levels of singing-induced dusp1 expression and little to no egr1 expression in songbird aDLM, parrot DMM, and hummingbird aDLM, indicate that these thalamic nuclei (as well as songbird Uva) did not acquire specialized co-expression of these two genes.

Second, we found that the auditory region L2 shows weak motor-driven dusp1 expression after singing in deaf animals. This finding still needs to be validated with electrophysiological recordings. The prior work that showed that zebra finch “field L” has neuronal firing preceding singing behavior [52] did not specify whether recordings were made from L2 (where dusp1 expression is induced) or the adjacent L1 and L3 (where dusp1 expression is not induced). We also found previously [34] that a small portion of L2, located above NIf and adjacent to the higher sensory part of PLN (including the anterior HVC shelf), shows both hearing- and movement-driven dusp1 expression; a similar result was obtained for PLN and egr1 (**Fig. 2C,G**; [34]). These results suggest that a narrow portion of the auditory pathway near NIf has comparably robust sensory-driven and motor-driven activity inside (dusp1) and outside (egr1) of L2. We hypothesize that this region of the avian forebrain could be a central hub where sensory and motor information are exchanged.

In summary, we discovered specialized motor-driven dusp1 expression in song nuclei of vocal learning birds. We suggest that during the evolution of vocal learning, some genes, such as egr1, maintained their functional properties from the cell types of the brain subdivision in which they were derived, whereas dusp1 might have been independently modified multiple times to take on new functional properties. It will be useful for future investigations to determine whether selection of differential dusp1 expression occurred in the vocal/speech brain areas of mammalian vocal learners, including humans.

## Materials and Methods

### Animals

We used 55 male zebra finches (*Taeniopygia guttata*; songbird), 10 male budgerigars (*Melopsittacus undulates*; parrot), 6 male sombre hummingbirds (*Aphantochroa cirrochloris*), 3 male Anna’s hummingbirds (*Calypte anna*), 3 male rufous-breasted hermits (*Glaucis hirsuta*; hummingbird), 7 eastern phoebes (*Sayornis phoebe*), and 7 ring doves (*Streptopelia risoria*). The zebra finches and budgerigars were from our breeding colonies at the Duke University Medical Center and Hokkaido University. The hummingbirds were captured from wild populations in Santa Theresa, Espirito Santo, Brazil (sombre hummingbird, rufous-breasted hermit) and in Riverside, California (Anna’s hummingbird). Some of the zebra finch and budgerigar sections, and all hummingbird sections are adjacent to sections of animals reported in our prior studies [22], [29], [34], [54], [94]. The adult singing time course, juvenile singing, flying, and seizure-induced groups of zebra finches and 1 silent and 2 singing budgerigars were all from this study. We used males, as they are typically the sex that has vocal learning and the associated forebrain song nuclei. All animal procedures conducted on animals bred in captivity were approved by the Institutional Animal Care and Use Committee of Duke University (protocol number: A107-08-04) or by the Committee on Animal Experiments of Hokkaido University (protocol number: No. 20 (9)). For animals from the field, procedures were approved by both Duke University (protocol number A322-03-09) and the University of California Riverside for the Anna's Hummingbird, the Instituto Brasileiro do Meio Ambiente E Dos Recursos Naturais RenovaÅLveis (#058/97-DIFAS) for the sombre and rufous-brested hermit hummingbird species, and by the Institutional Animal Care and Use Committee of the Rockefeller University for Eastern phoebes (protocol number: 08016; State permit: LCP316 and Federal permit: 09862 issued to Field research center at the Rockefeller University, Millbrook, New York).

### Behavior Experiments

#### Zebra finches

We examined 13 experimental groups: 1) silent non-singing controls; 2) birds that heard 30 min of song playbacks; 3) birds that sang for 30 min and heard themselves sing; 4) a continuous time course singing experiment (sang up to 180 min); 5) an acute time course singing experiment (0 [silent, from group 1], 15 min and 30 min of singing followed by 0 [from group 3], 30, 60, and 90 min of silence); 6) deaf birds that were silent; 7) deaf birds that sang for 30 min; 8) juvenile birds that were silent; 9) juvenile birds that sang for 30 min; 10) birds that hopped for 30 min in a rotating wheel; 11) birds that flew for 60 min; 12) birds that had a metrazole-induced seizure; and 13) birds that had a kainate-induced seizure.

For all experiments, birds were individually placed in sound-attenuated boxes overnight to reduce IEGs levels in the brain to baseline. On the next morning, the lights were turned on, and the birds were observed by video and audio for silent or singing behaviors (undirected singing). Behavior was recorded using Sound Analysis Pro (http://ofer.sci.ccny.cuny.edu/sound_analysis_pro; [95]) and by a digital video recorder. Silent controls (group 1; n  = 3) were kept without singing for 1 hour. The hearing song controls (group 2; n  = 3) were presented with digitally recorded songs through a speaker (three different songs, totaling 12 seconds, presented once every minute for 30 min) and did not sing, similar to a described protocol [38], [39]. The 30 min singing animals (group 3; n  = 5) were birds that spontaneously sang (without playbacks) for various amounts (60–314 sec, 25–138 song bouts) during the 30 min session. A song bout was defined as a continuous production of syllables followed by at least a 400 ms of silence. Singing duration was defined as the total amount of time spent producing song syllables. The transient time course experiment included the silent controls (0 time point, Group 1), birds that sang 15 min (group 5a; n  = 3) and 30 min (Group 2), and birds that either spontaneously stopped singing at 30 min or were influenced to stop singing by the presence of the investigator (next to an open sound-attenuation box) and were then sacrificed 30 min (group 5b; n  = 3) and 90 min (group5c; n  = 3) later (*i.e.* 60 and 120 min after the start of singing), following a previously described protocol [39], [68]. The continuous time course experiment also included the silent controls (0 time point, Group 1), birds that sang 15 min (group 5a; n  = 3) and 30 min (Group 2), and additional birds that were allowed to continue to sing for 60 and 180 min (group 4; n  = 3 each time point), at an average of 136.5 and 212.0 seconds of singing duration per hour, respectively.

For the deaf groups (groups 6 and 7; n  = 3 silent and 6 singing birds), animals were deafened as adults (102–113 days post-hatch; dph) by bilateral cochlea removal following a previously described protocol [94], [96]. Thereafter, they were placed in sound-attenuation boxes and on a given morning (2 weeks after deafening) after they sang a required amount of song (>83 sec of singing during the 30 min recording session, equivalent to >34 song bouts), their brains were dissected and prepared for *in situ* hybridization. The silent control deaf birds were treated the same way for the same time period, but did not sing at that time. Deafening surgery was verified by two measures: 1) Visual inspection of the shape of cochlea we pulled out; and 2) After recovery, lack of any behavioral response to vocalizations of other birds or of a startle response to loud clapping of our hands.

The juvenile groups (groups 8 and 9; n  = 3 silent and 5 singing birds) were from 41 to 48 dph, where the singers spontaneously sang various amounts (31–312 sec) of subsong alone during the 30 min session. For hopping animals (group 10; n  = 3), deafened birds were forced to hop in a rotating wheel for 30 min in the dark, as previously described [29]. For flying animals (group 11; n  = 3), birds were forced to fly in a room (∼35 square meters) for 60 min with 5 minutes rest every 15 minutes. For seizure birds (groups 12 and 13; n  = 3 metrazole-induced, n  = 3 kainate-induced), birds were injected intraperitoneally with either metrazole (pentylenetetrazole; SIGMA, St. Louis, MO) or kainate (kainic acid; Tocris Bioscience, Ellisville, MO) following a previously described protocol [53]. Seizure activity was monitored for 1 hour and additional drug was given if activity was not noted. Kainate-induced seizures started with mild tremor of wing tips and built up to chronic seizures with trembling of the whole body. The final dose was 60–80 mg/kg metrazole or 5–20 mg/kg kainate.

#### Budgerigars

We collected three experimental groups of animals, some from prior studies [22], [29], [34], [54]: 1) silent controls (n  = 3 total; n  = 2 from past studies); 2) birds hearing 30–60 min of warble song playbacks (n  = 4 total; all from past studies); and 3) birds singing for 30–60 min (n  = 3 total; n  = 1 from a past study). The experimental conditions were similar to that used for zebra finches, except that the animals were presented with a playback of recorded warbles for both the hearing and singing groups, as previously described [54], because it was very rare for us to observe budgerigars singing spontaneously in isolation without playbacks. For the singing group, we collected animals that sang 40–100 bouts of warble song (lasting 2–10 seconds on average).

#### Hummingbirds

We obtained brain sections from the same wild birds of prior studies [22], [29]: 1) silent controls (n  = 3 sombre hummingbirds, 1 Anna’s hummingbird and 1 rufous-breasted hermit; n  = 5 total) and 2) ∼30 min of singing (n  = 3 sombre hummingbirds, 2 rufous-breasted hermits, and 2 Anna’s hummingbirds; n  = 7 total). In brief, after 30 min of each behavioral condition, birds were caught in sugar-water (24%) baited cage traps. Silent controls were caught after waking and before the start of the dawn chorus. Singers were caught in the morning after they sang one or more song bouts per min for a ∼30 min period. We collected animals that sang ∼30–90 bouts of song (lasting 2–10 seconds on average). The animals were continuously observed with binoculars and a video camera from the time they awoke until sacrifice. Some of the individuals were captured the day before and placed in an outdoor aviary within its home territory, and observed and recorded from the time the animal woke up in the morning (i.e. observations started before sunrise) until sacrifice. If we lost sight of a bird, it was not captured.

#### Eastern phoebes

We collected brains from two groups of adult animals: silent controls (n  = 4) and singing (n  = 3). These phoebes were collected as nestlings from the field at the Rockefeller University Field Research Center in Millbrook, New York during the breeding season in June-July, and were hand-raised until 35–40 dph. After sexual maturity (260–280 dph), each bird was individually placed in a sound isolation chamber overnight, and was sacrificed after 45 min of silence or singing on the next morning. We recorded song behavior with a microphone in the box. The singing group spontaneously vocalized the innate ‘fee-bee’ song. We collected three animals that sang: 1) more than 100 songs in 45 min after lights were turned on; 2) more than 300 songs in 80 min; and 3) more than 100 songs (15 minutes singing duration) in 45 min. The sex of the birds was verified by gonad dissection after sacrifice. A separate study is being prepared on a more detailed analysis of singing-driven expression in the phoebe brainstem.

#### Ring doves

We collected two groups of animals: 1) silent controls (n  = 3 total all from our past dusp1 study [34]); and 2) birds that cooed alone for 30 min (n  = 4). The experimental conditions were similar to that used for zebra finches. The animals were kept in the sound isolation box overnight, and collected for groups depending on the planned conditions and their spontaneous behavior. Typically, ∼45 min after the lights came on in the morning, males in the vocalizing group generated coos. We collected animals that sang 30–70 bouts of coos (lasting 2–10 seconds each, or 2–5 coos/bout/min on average). Those that did not coo in the same time period were taken as the silent control group.

### *In situ* Hybridizations and Double Labeling

After each of the above behavioral sessions, birds were decapitated, and their brains were removed, embedded in OCT compound (Sakura Fine Technical, Tokyo, Japan), frozen and stored at –80°C. *In situ* hybridizations were performed as previously described [68], [97]. In brief, 12 µm frozen sections were cut in the sagittal or coronal planes. Corresponding sections of all birds of experimental comparisons of interest were fixed in 3% paraformaldehyde and processed for *in situ* hybridization with antisense ^35^S-UTP labeled riboprobes of zebra finch dusp1 or egr1, as described in [34] and [68], respectively. Hybridization temperature and washes were at 65°C for egr1 in all species and for dusp1 in zebra finches, and 60°C for dusp1 in all other species, due to a need for cross species hybridization. The 60°C temperature was determined by trial and error to obtain a maximum difference between background signal and dusp1-^35^S signals in Field L2 and/or song nuclei [34]. Due to the lower stringency hybridization, the background signal of *in-situ* hybridizations was a little stronger for some (for hummingbird, phoebe and ring dove). However, the sense strand of dusp1 showed the same low background signal as did the antisense (not shown). The hybridized sections were exposed to X-ray film (Biomax MR, Kodak, Rochester, NY) for 1–4 days, then dipped into autoradiographic emulsion (NTB2, Kodak), incubated for 1–3 weeks, processed with D-19 developer (Kodak) and fixer (Kodak), Nissl-stained with 2% cresyl-violet acetate solution (Sigma, St. Louis, MO), and coverslipped.

The double labeling *in situ* hybridization method for dusp1 and egr1 is described in detail in [34]. In brief, dusp1 expression was detected with a radioactive ^35^S-UTP labeled riboprobe and egr1 with a Digoxigenin (DIG)-UTP labeled riboprobe. The two probes were added simultaneously to the hybridization solution. After hybridization, the egr1-DIG label was reacted with an anti-DIG-alkaline phosphatase antibody and a BM purple (Roche, Indianapolis, IN) solution, and then the dusp1-^35^S signal detected by dipping slides in Ilford autoradiography emulsion (Ilford K5, polyscience; Kodak emulsion strips away the DIG signal). The emulsion was exposed for 1–2 weeks at 4°C, processed with D-19 developer (Kodak) and fixer (Kodak), and the slides coverslipped with mounting medium (VECTASHIELD with DAPI, Vector, Burlingame, CA). The egr1-DIG mRNA label is purple in color, located in the cytoplasm, and the dusp1-^35^S label appears as black silver-grains in the emulsion above the cell cytoplasm.

### Quantification and Statistics

Gene expression in song nuclei and other brain regions was quantified following a previously described procedure [68]. We used X-ray film of brain images that were digitally scanned from a dissecting microscope connected to a SPOT-III CCD camera with SPOT imaging software (Diagnostic Instruments, Sterling Heights, MI). Care was taken to use the same light settings across all images of an experiment. We used Adobe Photoshop CS3 (Adobe Systems, San Jose, CA) to measure the mean pixel intensities in the brain areas of interest from two or more adjacent sections on a 256 grey scale. To quantify and compare the relative amount of dusp1 versus egr1 expression among song nuclei of the singing or hearing animals relative to non-singing silent controls, we normalized the amount of dusp1 or egr1 expression in each song nucleus by the average amount in silent controls. Statistical differences were determined by repeated measures ANOVA for overall differences between silent controls and singing groups, followed by unpaired *t*-test among groups, and by ANOVA among song nuclei reacted with each gene. To examine the amount of singing as a variable, we performed a regression analysis on total time spent singing (in seconds)for each animal of the 30 min singing group versus the amount of dusp1 expression in each nucleus, and performed multiple regression analysis to compare regression lines between 2 to 3 groups of animals for each song nucleus. To perform the time course analysis, we performed repeated measures ANOVA if there were overall differences among time points followed by a Dunnett’s post test of each time point relative to the silent control for each song nucleus; a Dunnett’s test is designed to compare one control group relative to multiple other groups. For all test, significance was considered at p<0.05.

Double label cells were quantified using a previously described procedure [34]. We used a compound microscope at 60x magnification and Slidebook software (Olympus, Tokyo, Japan) to acquire images of the regions of interest. The total number of labeled cells (range 50–71, n  = 3 birds) within a given field from at least two adjacent sections was counted. Of this total, the subsets of single and double labeled dusp1 and egr1 cells were estimated and corrected with the Abercrombie equation (N = n(T/(T+D)), where N is the corrected number of the labeled cells, n the estimated number of the labeled cells, T the thickness of the section (12 µm), and D the mean diameter of the nuclei [98]. We only considered a cell labeled if we could find a clear nucleus counterstained by DAPI or lightly co-stained by the chromogenic background signal (purple/brown reaction product) associated with the DIG reaction product (for egr1) or ∼10 or more silver grains above the cell (for dusp1). DIG labeled cells were distinguished from the background by visual inspection. The chromogenic background created a shadow effect for all cell nuclei in the brain, including in areas where egr1 is known not to be expressed (lighter inside the nucleus; **Fig. 3D**, white arrows). These shadow labels and DAPI overlapped. We found it easier to use the shadow label in the same image settings as the real label to unambiguously locate individual cells. The average background number of silver grains per an average cell size area on the glass without tissue was 1–7. Thus, we set the background conservatively to be 10 grains as a criterion for gene induction. Since the number of labeled dusp1 and egr1 cells varied according to the amount of singing for song nuclei, hopping for motor areas, and hearing song for auditory regions, we had to normalize this behavioral variable to quantify the proportion of double labeled cells. To do so, for each brain region, we calculated the mean percentage of dusp1+, egr1+, and dusp1+/egr1+ relative to all labeled cells, and statistically compared the values within and across brain areas by ANOVA.

For the summary figure of expression (**Fig. 10**), we used singing birds for song nuclei, and hopping zebra finches and budgerigars or flying hummingbirds for the movement-activated areas [29]. For each calculation (song nuclei or non-vocal motor areas), signal intensity of brain areas where each gene was minimally up-regulated (dusp1 in NCM, egr1 in L2) was set to 0, and where it was up-regulated most was set to 1 (dusp1 in LMAN, NAO, VMN and egr1 in AreaX, MMSt, VAN for zebra finch, budgerigar, and hummingbird respectively). The other areas were calculated relative to these, and scaled from 0 to 1. Then the relative level of gene induction (L) was color-coded into 5 graded colors from white to green (L ≤0.05, 0.05< L ≤0.25, 0.25< L ≤0.50, 0.50< L ≤0.75, 0.75< L ≤1.0).

### Cloning and Sequence Analysis of Putative Dusp1 Regulatory Regions

To clone the dusp1 promoter regions, we extracted genomic DNA from either blood or brain sections of the following species: zebra finch (*Taeniopygia guttata*), owl finch (*Taeniopygia bichenovii*), star finch (*Neochmia ruficauda*), cherry finch (*Neochmia modesta*), Bengalese finch (*Lonchura striata domestica*), budgerigar (*Melopsittacus undulates*), blue-fronted Amazon (*Amazona aestiva*), sulphur-crested cockatoo (*Cacatua galerita*), sombre hummingbird (*Aphantochroa cirrochloris*), Anna’s hummingbird (*Calypte anna*), eastern phoebe (*Sayornis phoebe*), great-crested flycatcher (*Myiarchus crinitus*), rock pigeon (*Columba livia*), chicken (*Gallus gallus*), blue-breasted quail (*Coturnix chinensis*), Japanese mountain hawk-eagle (*Nisaetus nipalensis orientalis a.k.a. Spizaetus nipalensis orientalis*), and white-naped crane (*Grus vipio*). Then, we used forward (5′) and reverse (3′) primers that recognizes conserved regions based on the zebra finch and chicken genome alignments (UCSC genome database) in a PCR reaction. Since we were not sure which part of the sequence was conserved for each species, we tried several different primers within the conserved region. Primers that worked for each species were as follows: the forward 5′ oligo DNA primers were: 5'- GGCAGGTTTATTTAAGAAAAGAAA-3′ (pigeon), 5′- CAAAAATAAAGCAAGGAAATAGC-3′ (budgerigar), 5′-AGTGATTAAGTACACACTGCC-3′ (mountain hawk-eagle, Anna’s hummingbird), AAGTACACACTGCCACATGTG-3′ (zebra finch, eastern phoebe, great-crested flycatcher, white-naped crane), 5′-TACACACTGCCAYATGTGAT-3' (blue-fronted Amazon, sulphur-crested cockatoo, sombre hummingbird, blue-breasted quail), between -2696 to -2794 bp from start codon of chicken dusp1 gene; and the reverse 3′ primers were: 5′- TTGAAGGAGAAGAAGGAGCGG-3′ (sulphur-crested cockatoo, mountain hawk-eagle, blue-breasted quail), 5'-CACACCCGCAGGTTCACCAT-3′ (zebra finch, budgerigar, eastern phoebe, great-crested flycatcher, white-naped crane), 5′-AGTCGAGGACGAGGCACTG-3' (blue-fronted Amazon, sombre hummingbird, pigeon), 5′- GTTGCAGGAGCCGCGGATG-3′ (Anna’s hummingbird), between 0 and 135 bp from start codon of chicken dusp1. PCR conditions were 95°C for 4 min followed by 30 cycles of 95°C for 45 sec, 53°C for 30 sec, and 72°C for 3 min with Ex taq polymerase (Takara, Ohtsu, Japan), except for blue-fronted Amazon, eastern phoebe, great-crested flycatcher, white-naped crane, mountain hawk-eagle. The PCR condition for these other species were 94°C for 5 min followed by 30 cycles of 98°C for 10 sec, 60°C for 15 sec, 68°C for 3.15 min with primeSTAR GXL DNA polymerase (Takara). PCR products were examined on 1% agarose gels, extracted from the gels, ligated into the pGEM-T Easy plasmid (Promega), and transformed into XL-1 blue *E. Coli* cells. Plasmid DNA was isolated and the inserted cDNA was sequenced using vector specific primers. From these sequences, for each species, we then designed another set of primers to complete the insert sequence from the 5′ and 3′ ends (19–22 bp primers). In each species, to confirm that dusp1 was cloned, the sequences were BLAST searched against the UCSC genome database and homologies to zebra finch and chicken dusp1 coding and regulatory sequences were found. The Genbank accession numbers of our regulatory sequences are AB574425 for zebra finch, AB640892 owl finch, AB640893 star finch, AB640894 cherry finch, AB640895 Bengalese finch, AB574426 budgerigar, AB640886 blue-fronted Amazon, AB640887 sulphur-crested cockatoo, AB574427 sombre hummingbird, AB574428 Anna’s hummingbird, AB640888 eastern phoebe, AB640889 great-crested flycatcher, AB574429 rock pigeon, AB640896 blue-breasted quail, AB640890 white-naped crane, AB640891 mountain hawk-eagle.

For each species, we used Cluster Buster (http://zlab.bu.edu/cluster-buster/cbust.html) [99] to search for *cis*-regulatory elements and Tandem Repeat Finder (TRF; http://tandem.bu.edu/trf/trf.basic.submit.html) [100] for repetitive elements. In addition, we used RepeatMasker (Smit, AFA, Hubley, R & Green, P. RepeatMasker Open-3.0. 1996–2010<http://www.repeatmasker.org>) to find more complex repetitive elements such as retrotransposon sequences. The cloned sequences were aligned by DIALIGN software (http://bibiserv.techfak.uni-bielefeld.de/dialign/) [62] and displayed with Jalview software (http://www.jalview.org/) [101]. We then used the dialign generated fasta alignment file and the mega software (http://www.megasoftware.net/) [63] to generate phylogenetic trees with the maximum likelihood estimates. To calculate the percent of species with identical sequence among vocal learners and among vocal non-learners, we counted the number of species whose bases are identical to each other in each group. In each group, at least 2–3 vocal learners (pink) or 3 or more vocal non-learner lineages (grey) were used for the calculation. One species was selected as a representative of each lineage such as chicken for Galliformes (representative species have a star in **Fig. 9A**). This number was then divided by the number of representative species that have bases in the aligned genome sequence. We then averaged the percent identity of 10 consecutive base pair windows and plotted them in a graph. The difference was normalized by the standard deviation of each value.

The 3169 bp upstream sequence of zebra finch dusp1 we cloned is 98.9% identical to segments of an incomplete assembled dusp1 sequence from the draft zebra finch genome database (UCSC browser; [61]). Based on our analysis, we believe there was an error in the zebra finch genome assembly, placing a 100 bp gap in the promoter region and duplicating 200–1000 bp long segments of the promoter region in tandem due to the repetitive DNA (starting 279 bp 5' to the putative start codon; **Fig. 9**). Thus, for all comparisons, we used the sequences that we cloned, except for chicken, which appeared to have a correct assembly based upon the alignments we generated, without microsatellites.

### Nomenclature

We used the new avian brain nomenclature [102], [103] with modifications as discussed in several previous reports [29], [34], [45], [104], [105]. In brief, we relabeled the formally named hyperstriatum dorsale (HD) as mesopallium dorsale (MD) and the formally named hyperstriatum ventrale (HV) as mesopallium ventrale (MV), due to the presence of genes, such as FoxP1 and GluR1, whose expression is specialized in the mesopallium [42], [83]. Based on this definition, caudal mesopallium (CM) and nucleus avalanche (Av) and oval nucleus of the mesopallium (MO) of songbirds, the MO of parrots, and vocal nucleus of the anterior mesopallium (VAM) of hummingbirds are all within the ventral mesopallium. Abbreviations are in **Table 1**.

Some investigators equate songbird as a common names for the latin Passeriformes, with songbird as an order of birds that consist of both suborders oscines and sub-oscines. However, others use songbird to describe oscines only. Both usages are present in the published literatures. Thus, until phylogenetic analyses separates oscines and subosciene as two separate “orders”, we prefer the use of the term songbird as the common name for Passeriformes, and “suboscine songbird” and “oscine songbird” to refer to the two suborders.

### Figure Preparation

The above mentioned camera systems or a DFC490 CCD camera and Leica application suite (Leica Microsystems, Bannockburn, IL) were used for making pictures of the emulsion dipped slides. As described in [34], the photomicrographs were adjusted in Adobe Photoshop CS3. The levels function was used for all photomicrographs to expand the range of pixels. Images were further adjusted by the color adjustment function so that the signals in white had clear contrast to detect minor levels of gene expression for qualitative analysis. All images of the same gene in each species were adjusted in the same way to avoid modification in gene expression across groups.

## Supporting Information

Figure S1 **Lack of strong induction of dusp1 in movement-activated and other brain areas.** Darkfield images of *in situ* hybridization with dusp1 (**A**) and egr1 (**B**) in vocal areas and adjacent movement-activated areas of singing birds with long-term exposure of the emulsion. (**C**) Representative images of dusp1 expression in birds that had seizures induced by kainate injection. (**D**) Adjacent sections hybridized with egr1. The white smudge over the anterior ventral part of the section in D is an emulsion artifact, which did not affect the white radioactive signals. Scale bars  = 50 µm for (**A**) and (**B**), 1mm for whole brains in C and D, and 500 µm for high power images of song nuclei in (**C**) and (**D**).
(JPG)

Figure S2 **Dusp1 expression in MMSt of budgerigars. (A**) Darkfield image of *in situ* hybridization in MMSt of the striatum with dusp1 from a singing bird. (**B**) Bright field Nissl stain image of the same section. Arrows point to the larger cells in MMSt. Scale bar  = 200 µm.
(JPG)

Figure S3 **Hearing-induced dusp1 expression in budgerigar brain.** (**A**) Example darkfield images of *in situ* hybridizations with dusp1 from a silent control male budgerigar sitting still (no auditory stimulus) in the dark in a sound attenuation chamber (**A_1_**), and a male bird under the same conditions except that he heard playbacks of songs (**A_2_**). (**B**) Adjacent sections hybridized to egr1. These examples show that dusp1 is specifically induced in L2 (as well as Ov of the thalamus) and egr1 is induced in the adjacent NCM, CM, and CSt (higher order auditory neurons) due to hearing song; neither gene is induced in MMSt, NAO, and MO (song nuclei) by hearing song, summarizing our past findings. White, gene expression, mRNA signal. Red, cresyl violet stain. Sections are sagittal. Scale bar  = 2 mm.
(JPG)

Figure S4 **Comparison of dusp1 expression in song nuclei of three hummingbird species.** (**A_1–3_**) sombre hummingbird, (**B_1–3_**) rufous-breasted hermit, and (**C_1–3_**) Anna’s hummingbird. Sections are from male birds that sang for about 30 minutes. Yellow lines, vocal areas where dusp1 was up-regulated. Scale bar  = 500 µm in C_1_ (applies to A_1_,B_1_,C_1_), C_2_ (applies to A_2_,B_2_,C_2_), and C_3_ (applies to A_3_,B_3_,C_3_).
(JPG)

Figure S5 **Hypothesized molecular interactions of dusp1 and egr1 in the brain.** Models are based on the known molecular pathway of these genes in cultured cells [36], [71]–[73], *in-vivo* regulation in the brain [34], and this study**.** (**A**) Model of dusp1 expression inhibiting egr1 expression in cell culture experiments is consistent with our findings in sensory-input neurons of the thalamus and telencephalon. (**B**) Model of high egr1 expression in the absence of high dusp1 from cell culture experiments is also consistent with our findings in higher order sensory neurons and motor areas. (C) Model of high dusp1 and egr1 expression in song nuclei, highlighting parts of this pathway (? mark) where genetic changes in dusp1 regulation and function could best explain the results found in song nuclei of this study.
(JPG)
