# Supplementary figures and images for: Specialized Motor-Driven dusp1 Expression in the Song Systems of Multiple Lineages of Vocal Learning Birds
Source: PLoS One. 2012 Aug 2;7(8):e42173. doi: 10.1371/journal.pone.0042173 (PMC3410896; doi:10.1371/journal.pone.0042173)

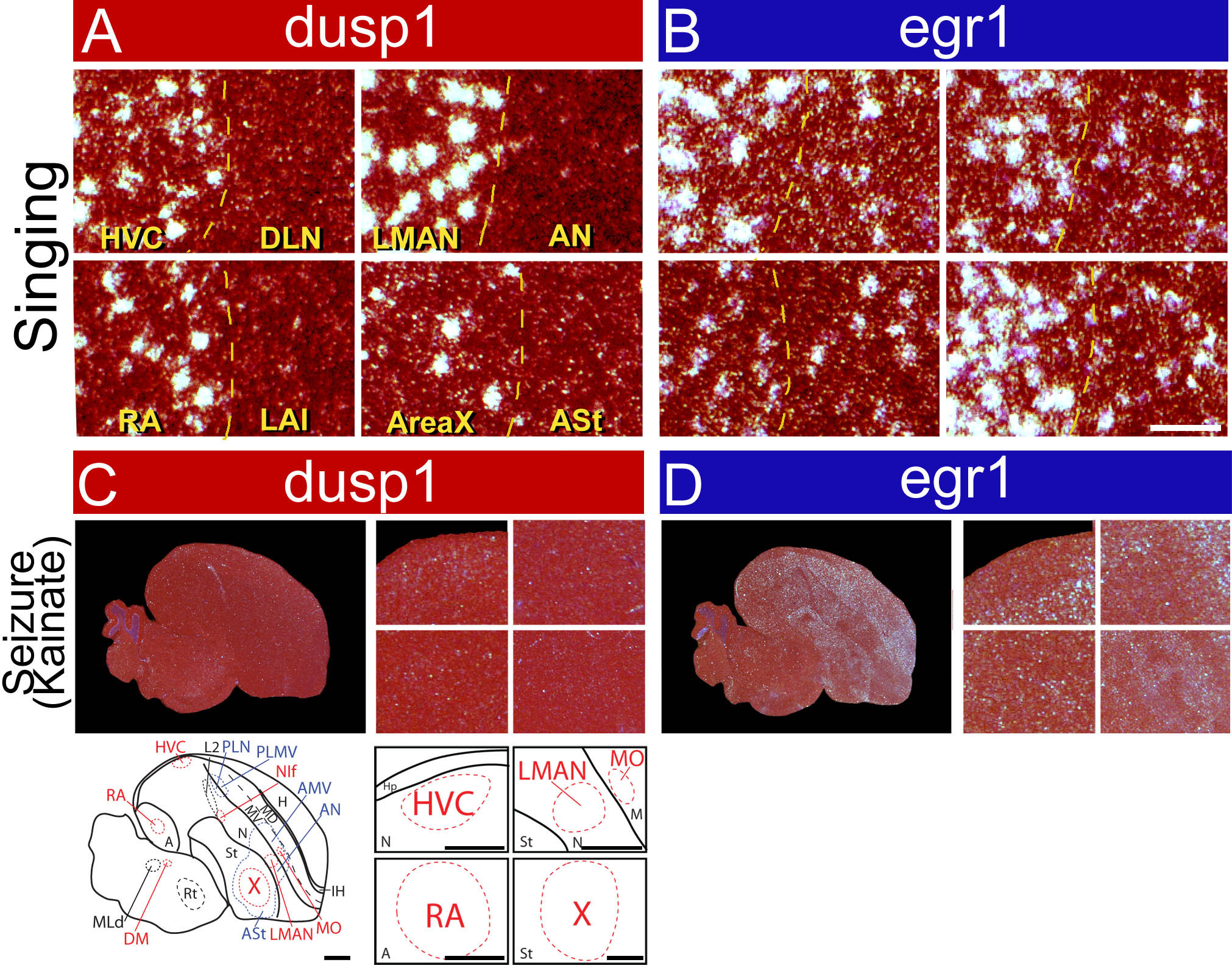

Supplement: Figure S1 — Lack of strong induction of dusp1 in movement-activated and other brain areas. Darkfield images of in situ hybridization with dusp1 (A) and egr1 (B) in vocal areas and adjacent movement-activated areas of singing birds with long-term exposure of the emulsion. (C) Representative images of dusp1 expression in birds that had seizures induced by kainate injection. (D) Adjacent sections hybridized with egr1. The white smudge over the anterior ventral part of the section in D is an emulsion artifact, which did not affect the white radioactive signals. Scale bars = 50 µm for (A) and (B), 1mm for whole brains in C and D, and 500 µm for high power images of song nuclei in (C) and (D). (JPG) [file pone.0042173.s001.jpg]

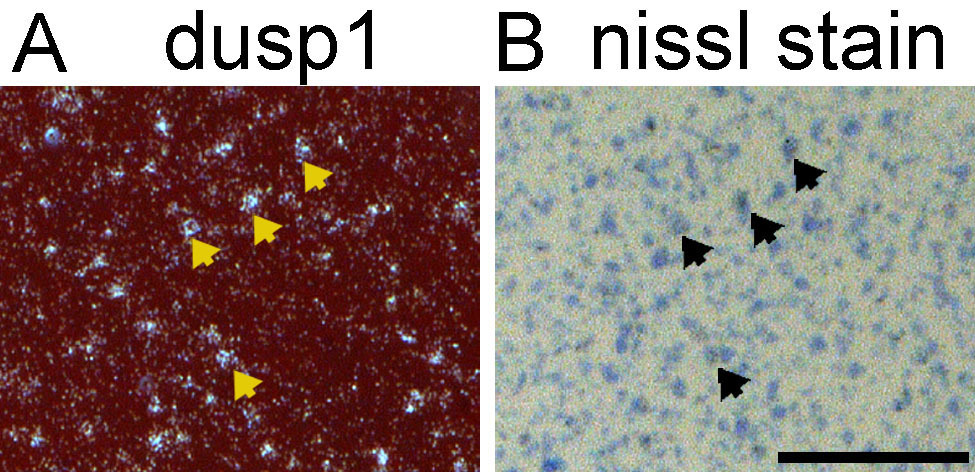

Supplement: Figure S2 — Dusp1 expression in MMSt of budgerigars. (A) Darkfield image of in situ hybridization in MMSt of the striatum with dusp1 from a singing bird. (B) Bright field Nissl stain image of the same section. Arrows point to the larger cells in MMSt. Scale bar = 200 µm. (JPG) [file pone.0042173.s002.jpg]

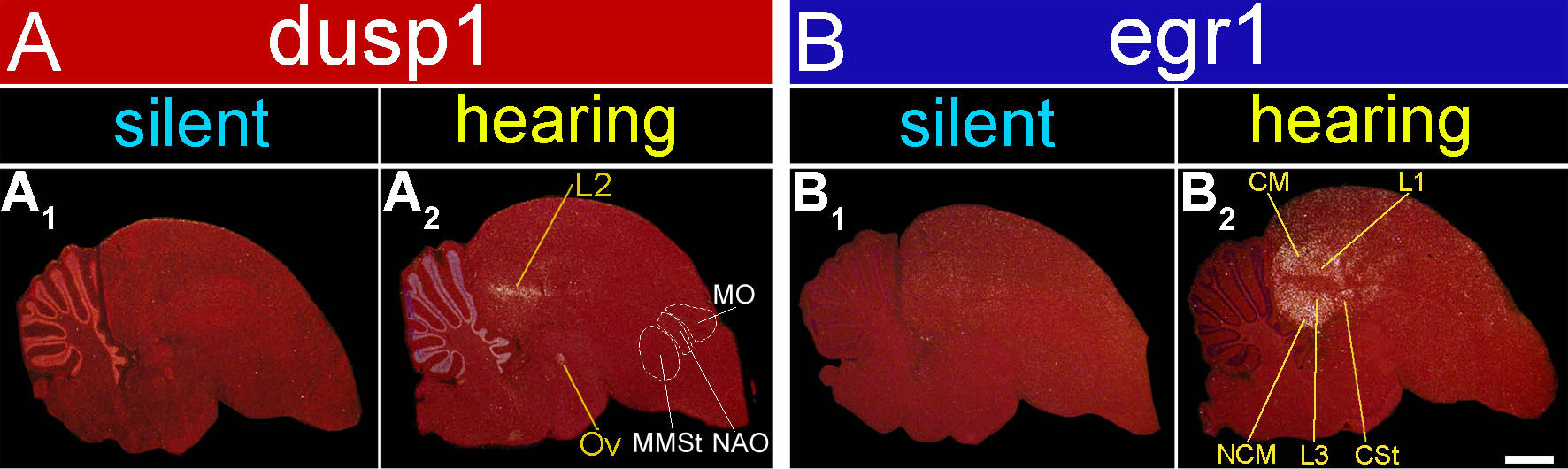

Supplement: Figure S3 — Hearing-induced dusp1 expression in budgerigar brain. (A) Example darkfield images of in situ hybridizations with dusp1 from a silent control male budgerigar sitting still (no auditory stimulus) in the dark in a sound attenuation chamber (A1), and a male bird under the same conditions except that he heard playbacks of songs (A2). (B) Adjacent sections hybridized to egr1. These examples show that dusp1 is specifically induced in L2 (as well as Ov of the thalamus) and egr1 is induced in the adjacent NCM, CM, and CSt (higher order auditory neurons) due to hearing song; neither gene is induced in MMSt, NAO, and MO (song nuclei) by hearing song, summarizing our past findings. White, gene expression, mRNA signal. Red, cresyl violet stain. Sections are sagittal. Scale bar = 2 mm. (JPG) [file pone.0042173.s003.jpg]

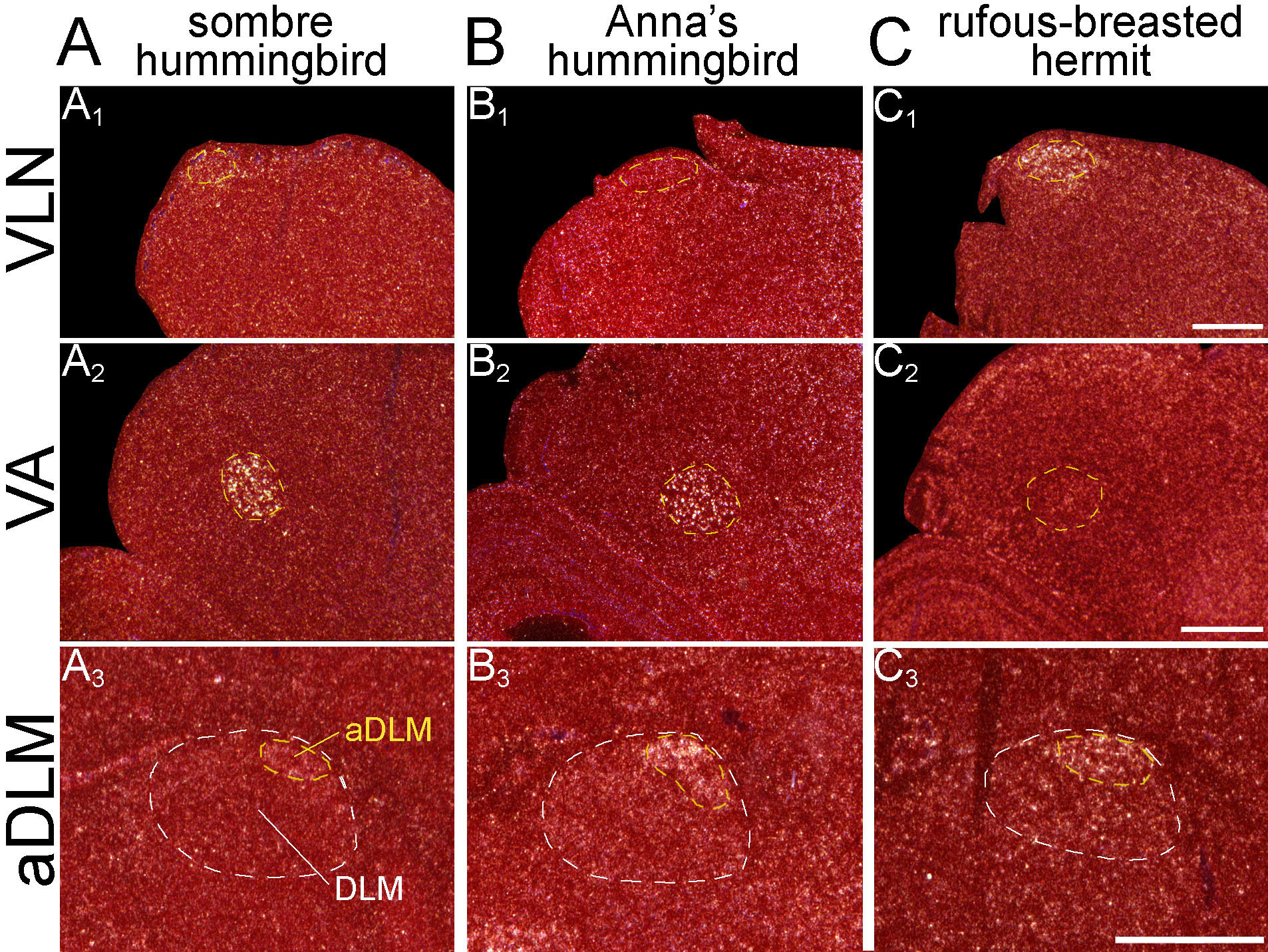

Supplement: Figure S4 — Comparison of dusp1 expression in song nuclei of three hummingbird species. (A1–3) sombre hummingbird, (B1–3) rufous-breasted hermit, and (C1–3) Anna’s hummingbird. Sections are from male birds that sang for about 30 minutes. Yellow lines, vocal areas where dusp1 was up-regulated. Scale bar = 500 µm in C1 (applies to A1,B1,C1), C2 (applies to A2,B2,C2), and C3 (applies to A3,B3,C3). (JPG) [file pone.0042173.s004.jpg]

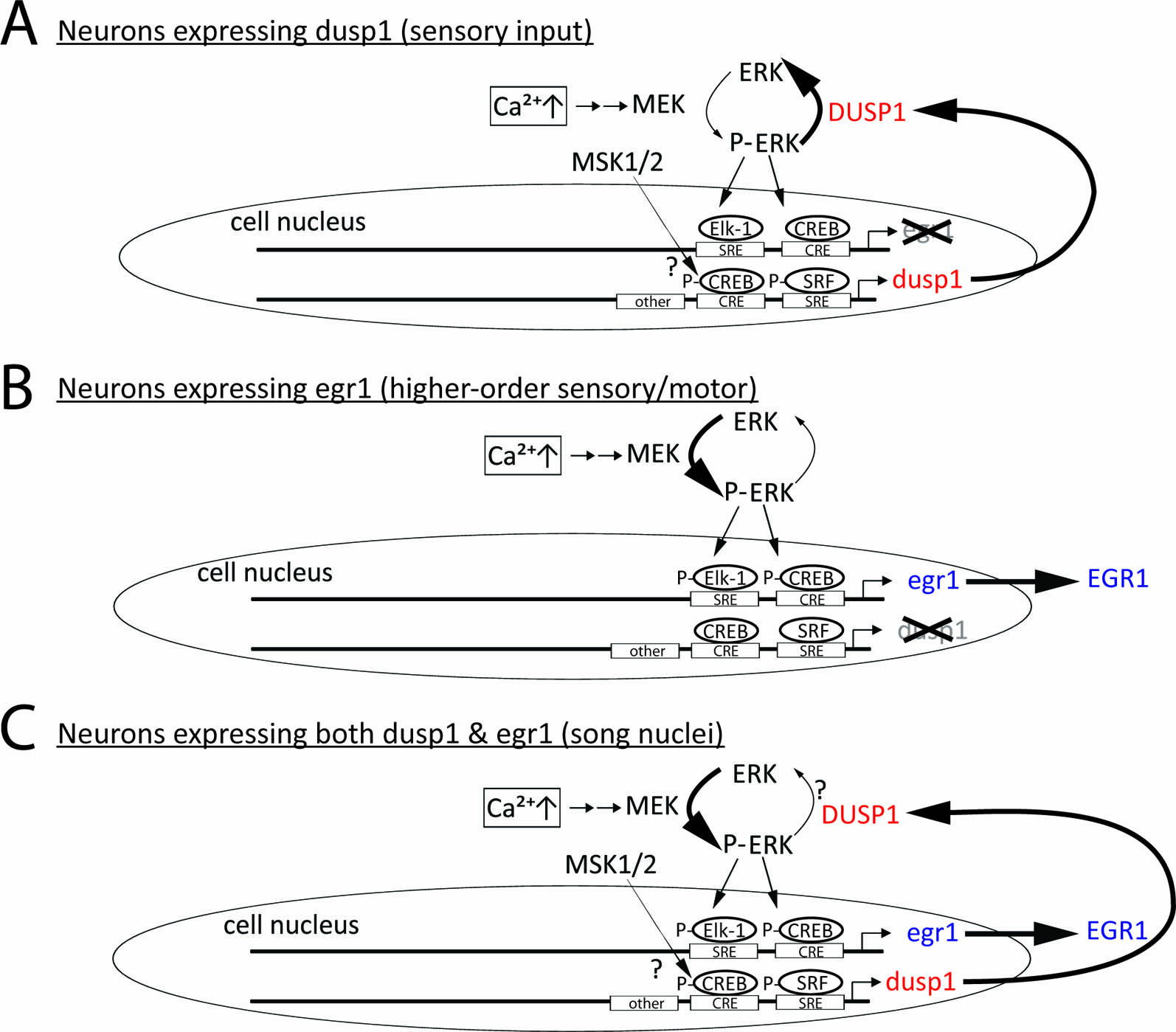

Supplement: Figure S5 — Hypothesized molecular interactions of dusp1 and egr1 in the brain. Models are based on the known molecular pathway of these genes in cultured cells [36], [71]–[73], in-vivo regulation in the brain [34], and this study. (A) Model of dusp1 expression inhibiting egr1 expression in cell culture experiments is consistent with our findings in sensory-input neurons of the thalamus and telencephalon. (B) Model of high egr1 expression in the absence of high dusp1 from cell culture experiments is also consistent with our findings in higher order sensory neurons and motor areas. (C) Model of high dusp1 and egr1 expression in song nuclei, highlighting parts of this pathway (? mark) where genetic changes in dusp1 regulation and function could best explain the results found in song nuclei of this study. (JPG) [file pone.0042173.s005.jpg]
